# Sustainable CO_2_ valorization for PHB production towards circular economy: metagenomic insights on enriched indigenous microbial cultures

**DOI:** 10.1038/s41598-025-26791-7

**Published:** 2026-01-10

**Authors:** Isha Bodhe, Velvizhi Gokuladoss

**Affiliations:** 1 School of Biosciences and Technology, Vellore Institute of Technology, Vellore, India; 2 CO2 Research & Green Technologies Centre, Vellore Institute of Technology, Vellore, India

**Keywords:** CO_2_ sequestration, Circular bio-economy, Polyhydroxybutyrate, Volatile fatty acids, Microbial enrichment, Metagenomics, Biological techniques, Biotechnology, Microbiology

## Abstract

This study developed resilient mixed microbial cultures (MMCs) from diverse indigenous sources _2_ through strategic pretreatment and enrichment, robust conversion of CO_2_ and bicarbonate into volatile fatty acids (VFAs) and polyhydroxybutyrate (PHB) was achieved. This article establishes a sustainable biotechnological platform, transforming waste CO_2_ into valuable bio-products and biodegradable polymers, thereby bridging a circular bioeconomy approach. In Phase I, CO_2_ was converted into VFAs (2.017, 2.307, 3.243, and 3.467 g/L) by anaerobic MMC and PHB (3.2% (0.082 g/L)) by aerobic cultures, respectively.The reduction in pH was observed to 3.45 ± 0.22 due to pretreatment methods, indicated acetogenic dominance. In phase II, VFA was recirculated for PHB production (6.5 ± 0.24%) and molecular confirmation was done using FTIR and NMR.ted The anaerobic MMC achieved a CO_2_ reduction efficiency of 70 ± 1.78%, while aerobic cultures showed 45 ± 1.26%, contributing to the production of biodegradable PHB, thus closing loop towards circular bio-economy. This study also shows carbon balance and thermodynamic feasibility of converting CO₂ into PHB via microbial fermentation, highlighting the energy requirements and process efficiency. Metagenomic analysis of microbial consortia, using 16S rRNA (V3-V4) sequencing, identified key bacteria for CO_2_ bioconversion. Anaerobic genera like *Pseudomonas* and *Halomonas* dominated VFA production, while aerobic bacteria such as *Brevundimonas, Achromobacter* were predominant in PHB synthesis. KEGG analysis predicts genetic pathways for CO_2_ fixation.

**Supplementary Information:**

The online version contains supplementary material available at 10.1038/s41598-025-26791-7.

## Introduction

Environmental issues, food security, climate change, etc. have an impact on the Sustainable Development Goals (SDG). A sustainable strategy to address these global concerns is the shift from a fossil fuel-based economy to a bioeconomy. In particular, 395 million tons (MT) of fossil fuel-based plastic production releases > 850 MT of CO_2_, or 2% of the world’s total CO_2_ emission, while using 5–7% of the world’s oil supply^1^. Since the procedures utilised historically operate a linear economy centred on mining raw materials and transforming them into useable goods, the majority of waste is dumped openly at the end of the life cycle, potentially adding more than one gigawatt-hour of CO_2_ equivalent^2–4^. Most polymers have a linear life however, the traditional plastics life is mostly linear, 79% is dumped into landfill sites or leaked to the environment, 12% is incinerated and recycled (9%). However the alternative i.e. bioplastics are degradable and/or recycled at their end of life (EOL) by using the carbon–neutral energy. Due to their perceived sustainability and circularity the bioplastic is gaining more importance. The European Union (EU) has been promoting an economic model called the circular economy (CE) thus discouraging the traditional make, use, and throw system. In a circular economy, the goal is to keep materials in use for as long as possible. This can be done by designing products for easy repaire or reuse, by recycling materials, and by using renewable energy sources. The circular economy also aims to reduce waste by eliminating toxic chemicals and designing products that create minimal waste. The adoption of a circular bioeconomy (CBE) represents a critical approach to achieving sustainable waste management and fostering a future built on environmental responsibility. CBE emphasizes the cascading utilization of biological waste materials, transforming them into bio-based products and materials. These products are then designed to participate in a closed-loop system, enabling sharing, reuse, remanufacturing, and recycling. Alternatively, they can safely returned to the biosphere through well-established organic and nutrient cycles^5,6^.

Ecosystem stores carbon naturally through biological sequestration. Six routes allow microbes to incorporate CO_2_, although the Calvin-Benson-Bassham (CBB) pathway is most common. Due to the environmental adaptability, lower sterility requirements, and cost-effectiveness, mixed microbial cultures (MMCs) with significant microbial diversity are outperforming pure bacteria. Syngas-fermenting bacteria use the Wood-Ljungdahl pathway (WLP) to create acetyl coenzyme-A (CoA), a crucial precursor to biomass and fermentation^7^. Polyhydroxybutyrate (PHB), an energy-storing material found in microorganisms, is a possible substitute for petrochemical polymers. PHB-producing bacteria with metabolic diversity can effectively synthesize PHB using various carbon rich biorefineries under the right fermentation conditions^8^. Despite being energetically non-spontaneous, PHB formation serves as carbon storage under stressful conditions. PHB offers several additional advantages beyond biodegradability, including biocompatibility for medical and pharmaceutical applications, thermoplastic processability, and lower greenhouse gas emissions relative to petroleum-based plastics, making it a strong candidate for sustainable material substitution^9,10^.

Carbon capture and sequestration involves utilising CO_2_ from emission sources and storing it in various geological or biological formations. Current CO_2_ absorption systems often struggle to keep pace with increasing anthropogenic GHG emissions^11^. Using MMCs and the major acetogenic fermentation processes, biological CO_2_ valorisation can produce volatile fatty acids (VFAs) and byproducts in various biorefineries. The CO_2_-to-X pathway is a reliable mechanism to convert CO_2_ into useful compounds, especially by feeding CO_2_/CO gases to microbial cultures. Anaerobic homoacetogens ferment inorganic H_2_/CO_2_ and CO into VFAs under rigorous conditions. VFA can be biologically converted into biopolymers, thus feeding the single and double phases fermentation processes^12^.

Anaerobic conversion of H_2_/CO_2_ to volatile fatty acids (VFAs) is thermodynamically favorable and can nearly reach theoretical yields. However, two main challenges limit gas fermentation: the low solubility of H_2_ at 21 °C and 1 barr (1.62 mg/L) and the toxicity of VFAs, with acetic acid inhibitors at 5–12 g/L. These factors reduce VFA concentration and productivity. Additionally, high maintenance costs for sterile reactors and complex VFA recovery hinder the processes ^12^. Unlike CO_2_-metabolizing bacteria with limited PHB synthesis, sugar- or oil-consuming microbes like *Rhodococcus* can accumulate up to 80% PHB^13^. To boost CO_2_-driven PHB production, researchers explore two-stage bioprocesses combining bioconversion and electrochemistry. Although PHB production is costly due to expensive substrates and slow growth, mixed microbial cultures (MMCs) and VFAs from waste can improve feasibility. Acetate and butyrate favor hydroxybutyrate monomers; propionate and valerate favor hydroxyvalerates^14^. Studies show homoacetogens in animal feces convert H_2_/CO_2_ and CO to organic acids via Wood-Ljungdahl and acetyl-CoA pathways. These acetogens are commonly found in monogastric herbivores like rabbits and horses, aiding digestion and fermenting syngas effectively^15^. Thus, this study utilizes chicken and rabbit fecal microbiomes rich in homoacetogenic genera including *Clostridium, Thermoanaerobacterium, Bacillus*, *Proteiniphilum*^16^, and *Fastidiosipila*^17^.

Recent efforts to improve CO_2_-driven bioplastic production have explored several avenues. For example, Fedorova et al. (2023) focused on optimizing bioreactor conditions to overcome H_2_ mass transfer limitations, achieving high VFA productivity in a hollow-fiber membrane system^18^. Concurrently, Nogle et al. (2022) investigated the use of genetically engineered pure cultures of *Clostridium autoethanogenum* to enhance the conversion of syngas directly to PHB precursors^19^. While these approaches have advanced the field, they often rely on sterile conditions and highly controlled systems, which increases the operational costs. Furthermore, most studies have concentrated on inocula from conventional sources like anaerobic sludge^20^, leaving alternative, potentially more potent microbial consortia underexplored. A critical gap remains in identifying and enriching robust, indigenous mixed microbial cultures (MMCs) that do not require strict sterility and can efficiently co-utilize both CO_2_ and bicarbonate, a more soluble inorganic carbon source.

**Fig. 1:**
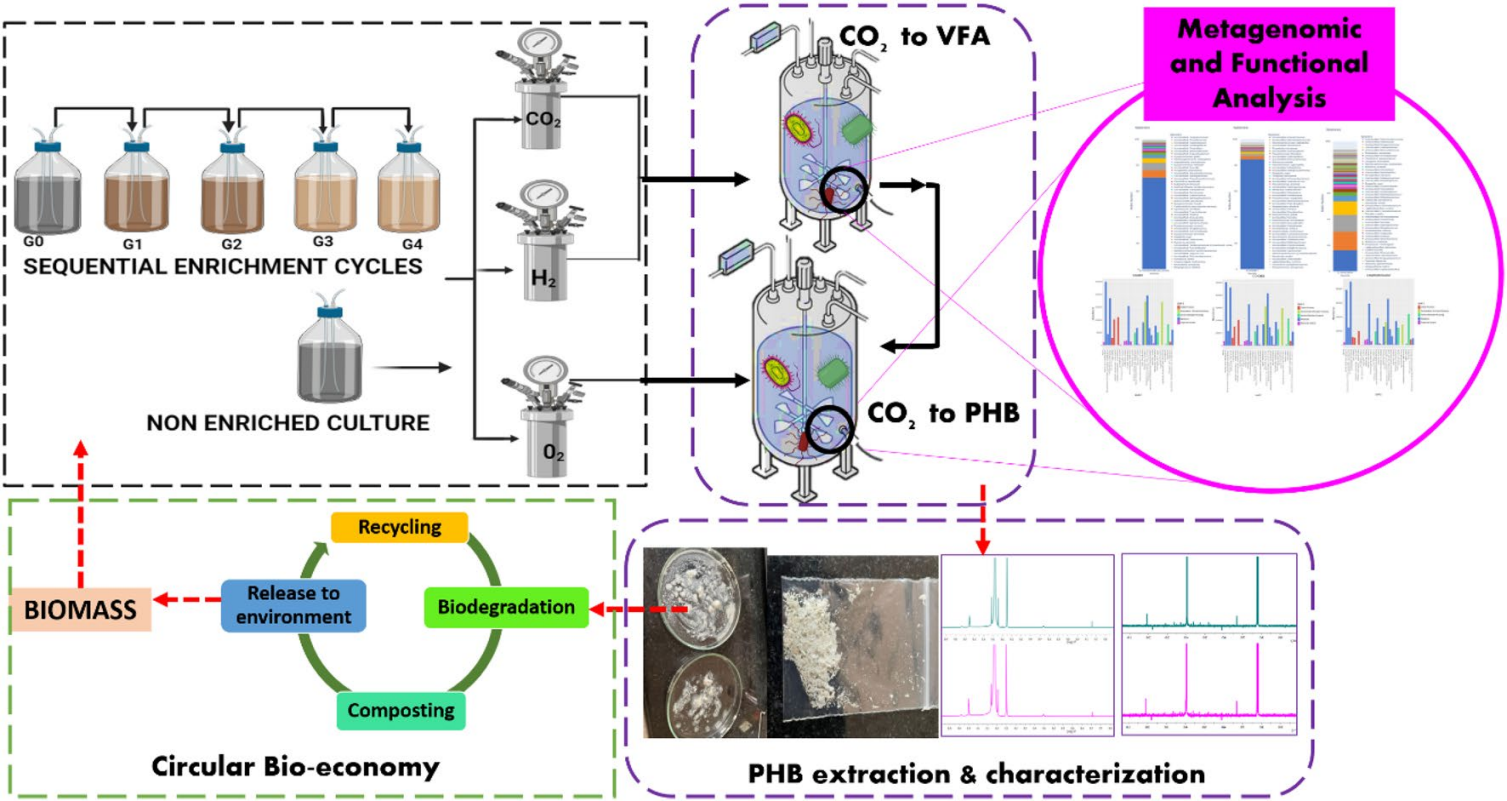
The schematic experimental work flow.

To address the aforementioned gap, this research uniquely identifies and enriches resilient, mixed microbial cultures from diverse indigenous sources (anaerobic sludge- AC, rabbit-RF, and chicken-CF faeces) as potent biocatalysts for efficient CO_2_ uptake. The novel pretreatment and gradual enrichment strategies to enhance their inorganic substrate utilisation. This work meticulously evaluates biocatalyst compatibility for improved PHB production during gas fermentation, concurrently analysing CO_2_ absorption enhancement. A rigorous carbon mass balance precisely quantifies CO_2_ conversion to VFAs and PHB, establishing a clear link to circular bioeconomy principles. Furthermore, integrating carbon mass balance, thermodynamic modelling, and 16S rRNA metagenomic analysis validated CO_2_ and bicarbonate as feedstocks, elucidating key microbial taxa and metabolic pathways crucial for carbon capture and sustainable bioplastics production. The schematic experimental flow is shown in Fig. 1.

## Results

### Pretreatment of homoacetogenic autotrophs

The three anaerobic MMCs were pretreated by heat and acid, the treated culture observed a pH reduction from 7.22, 8.74 and 7.62 to 5.97, 3.45, and 3.62 in AC, RF and CF respectively. The decrease in pH infers the dominance of anaerobic homo-acetogenic bacteria. Compared to the untreated culture (VFA—17.89 g/L, 12.37 g/L and 14.14 g/L in AC, RF and CF, respectively), the pretreated culture revealed higher VFA production of 22.48 g/L, 25.71 g/L and 27.62 g/L in AC, RF and CF respectively before enrichment. Studies reported that the pretreatment of the parent MMC encouraged the shift of its routine metabolic pathway towards acidogenesis and increase the production of H_2_ and prevent the competitive growth and coexistence of other H_2_ consuming organisms^21^. Homo-acetogens being obligate anaerobes, these bacteria rely on Wood-Ljungdahl Pathway as their main resource of energy conservation, cell carbon synthesis, and the synthesis of acetyl-CoA from CO_2_, which yields acetate as the main byproduct. Numerous microbes, such as homoacetogenic bacteria and the methanogenic Archaea, follow the same route. Also acetogens are spore forming communities; thus stressful conditions of pretreatment methods eliminate methanogens^21,22^. Pretreatment of homoacetogenic bacteria in anaerobic sludge, which contains both acetogens and competing methanogens, is crucial for efficient biohydrogen and alcohol production. Methanogens, which convert CO_2_ to CH_4_, cause carbon loss. Heat, acid, or alkali treatments can selectively eliminate methanogens, exploiting acetogen spore formation. This allows for enhanced CO_2_ fixation into acetyl-CoA, a precursor for valuable products like acetic acid, ethanol, and butanol^21–23^.

### Enrichment of anaerobic MMC producing VFA

**Fig. 2:**
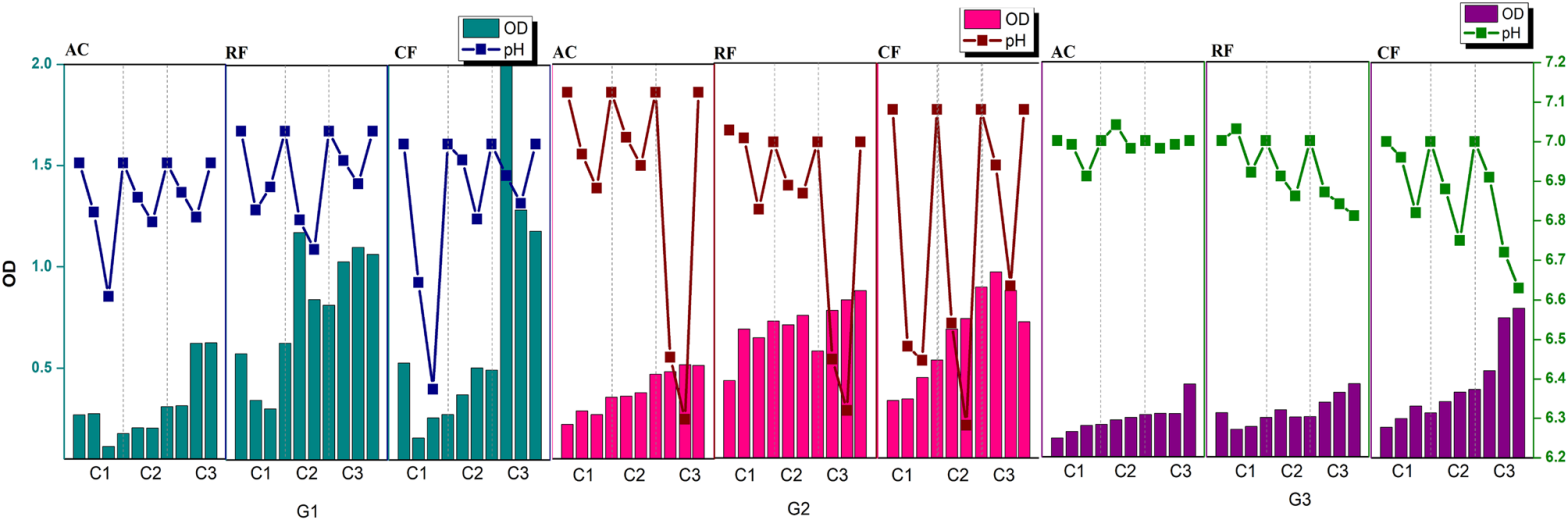
The graphical demonstration of G1, G2 and G3 showcasing the pH and OD trends for VFA production towards sodium bicarbonate consumption, for three MMC, i.e., anaerobic sludge (AC), Rabbit faeces (RF), and Chicken faeces (CF).

Following the pretreatment, the cultures were subjected explicitly to enrichment towards the headspace gas mixture (H_2_: CO_2_ (80:20) v/v) to promote the CO_2_ utilization. It was observed that the pretreatment and enrichment procedure increased the rate of CO_2_ consumption. The generations were labelled with respect to glucose and bicarbonate ratio such as G0–6:0, G1–4:2, G2-2:4, G3–0:6, and G4–CO_2_: H_2_ purging (20:80) along with 6 g/L bicarbonate. During enrichment, the growth of homoacetogens was presumed to account for the reduction of headspace gas, which might have been utilized for their development and metabolism. During the start of each cycle the pH in each culture was maintained at 7, which then reduced to 5.97 for AC, 3.45 for RF and 3.62 for CF in G0, 6.73 for AS, 6.14 for RF and 6.38 CF in G1; approximately ~ 6.3 for AC, RF and CF in G2 and 6.98 for AC, 6.63 for CF, and 6.81 for RF in G3 (Fig. 2). It was observed that cultures have higher buffering capacity given the culture conditions the findings also indicates that the culture medium becomes acidic due to the anabolism of VFAs. The slow change in organic to inorganic carbon source in the enrichment process helps to reduce the lag phase thus maximising the acclimation process^24,25^. The pH has a significant impact on the biocatalyst’s enzymatic activity. According to the graphs, all the generations represent an estimated rise in OD_600_ in the presence of bicarbonate ions and the preservation of pH equilibrium. Thus, after the enrichment of the three MMCs, the pretreated AC, CF, and RF were mixed with studying the behaviour of blended MMC. CO_2_ sequestering capabilities were observed to be higher in enriched culture compared to unenriched culture as control.

The rise in the percentage of bicarbonate reduction for every cycle was observed, for which titration was used to assess the concentration of bicarbonates and carbonates. An average of 45.68% in AC, 50.92% in CF, and 53.8% in RF bicarbonate reduction was observed during G1. In the case of G2, bicarbonate reduction was 46.1% in AC, 52.4% in CF, and 54.23% in RF. This could be attributed due to the microbial community being accustomed to inorganic carbon. A 49.6%, 53.5%, and 55.4% reduction in bicarbonate concentration was observed during G3 of AC, CF, and RF, respectively (Fig. 2). This signifies the utilisation of bicarbonate as an inorganic carbon substrate for bacterial growth and metabolism. Higher pressure ensures bicarbonate dissolution, whereas at lower pH, CO_2_ gas escapes in the head space. Thus, increasing the pressure on the system had an added benefit during enrichment, where high pressure conditions were automatically developed^26^.

### Enrichment of aerobic MMC producing PHB

**Fig. 3:**
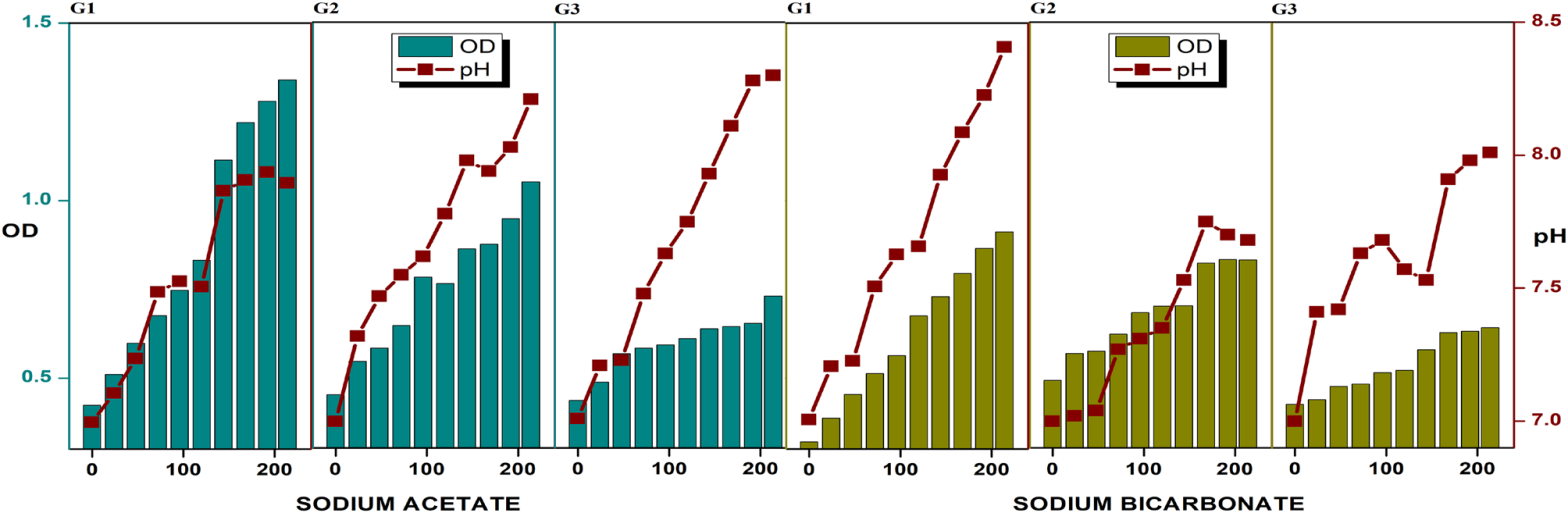
The graphical demonstration of G1, G2 and G3 showcasing the pH and OD trends for PHB production towards sodium bicarbonate and sodium acetate consumption.

The finding demonstrates the total reliance of microorganisms on sodium acetate and sodium bicarbonate as their alternate carbon source instead of glucose. Enrichment will shorten the lag phase for microorganisms to adapt to an environment where the carbon sources are organic acids (such as acetic acid, butyric acid) derived from biological sources (for VFA-PHB) and dissolved CO_2_ (for CO_2_-PHB). No pretreatment was done during this enrichment of aerobic MMC and the nitrogen-limiting condition of selective enrichment media was used. Initially, in G1- both cultures with the feed of 2 g/L sodium acetate and sodium bicarbonate separately showed enhanced buffering capacity. After G0, no significant pH shift was observed in the case of VFA-PHB and from 7 to 7.24 was seen in the case of CO_2_-PHB. A pH of 7.64 and 7.89 was observed, and the OD_600_ was 1.12 and 1.24 at the end of G1 with sodium acetate-MMC and sodium bicarbonate-MMC, respectively. In G2, the pH of 7 was maintained at the start of the cycle, which then increased to 7.71 and 8.52 in sodium acetate-MMC and sodium bicarbonate-MMC at the end of G2, with an increase in OD_600_ from 0.23 to 1.2 and 0.28 to 0.702, respectively. In G3, due to the higher concentrations of acetate ions, the pH has increased from 7 to 8.08, along with the increase in OD_600_ from 0.212 to 0.618. Similarly, due to the rise of bicarbonate ions, pH 7 increased to 8.34 with the increase in OD_600_ from 0.198 to 0.564 (Fig. 3). The addition of sodium acetate every 72 h causes a slight increase in the pH, as the hydrolysis of sodium acetate is mildly basic. During the enrichment, every sample was also characterised in pH, TSS, VSS, COD, and PHB content after the enrichment process. Biomass at the end of the enrichment reached a concentration of 182.67 mg TSS/L , as shown in Table 1, for sodium acetate as a carbon source and 284.67 mg TSS/L for sodium bicarbonate as carbon source (Table 1, Fig. 3).

**Table 1:** Physical characteristics of the enriched PHB inoculum.

	Sodium acetate	Sodium bicarbonate	CO_2_:H_2_:O_2_
TSS (mg/L)	182.67	284.67	151.63
TFS (mg/L)	160.0	182.67	147.79
VSS (mg/L)	22.67	72.2	25.4
PHB accumulation	4.1%	5.6%	3.2%
Biomass content (g/L)	4.45	4.21	2.55

A comparative study of enrichment with sodium acetate and sodium bicarbonate as sole carbon sources was done. Both acetate and bicarbonate ions, produced during the dissolution of sodium acetate and sodium bicarbonate (weak acids), are conjugate solid bases, thus making the medium weakly basic^27^. At the end of enrichment, the biomass content was calculated to be 4.45 and 4.21 g/L with sodium acetate and sodium bicarbonate as carbon sources, respectively (as shown in Table 1). Thus, it is inferred that the requirement of acetate ions is twice as high as that of bicarbonate ions to be incorporated into organic acids and lipids^25^.

### CO_2_ reduction for VFA and PHB production

**Fig. 4:**
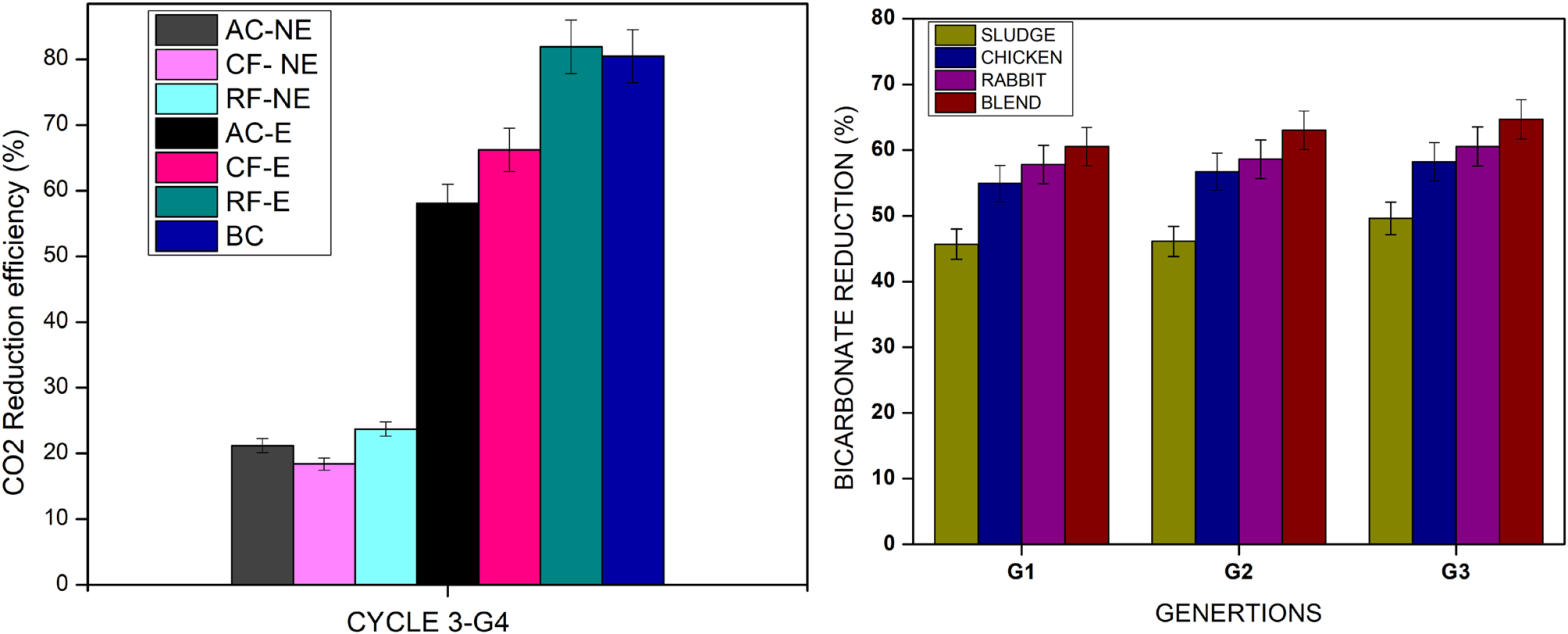
(**a**) The bicarbonate reduction pattern by the MMC by enriched (E) and non-enriched (NE) MMC (**b**) The comparative plot of CO_2_ reduction efficiency pattern by enriched (E) and non-enriched (NE) MMC.

During the transition from organic carbon (glucose) to inorganic carbon (bicarbonate and CO_2_), the CO_2_ consumption rates of the bacteria were rigorously evaluated. Initially, the 24 h CO_2_ consumption rates in AC, CF, and RF were observed to be 1.6%, 1.4%, and 25.4%, respectively. This process was carried out in a serum bottle, tested to withstand 2 barr of pressure, where the pressure built up at the time of CO_2_ sparging aided in the maximum dissolution of CO_2_:H_2_ gas. This built-up headspace pressure was reduced over time, suggesting bulk dissolution of gases via homoacetogens. To investigate the impact of different culture types and enrichment towards CO_2_ consumption efficiency, a two-way ANOVA was conducted, comparing Culture Type (AC, CF, RF) and Enrichment Status (non-enriched vs. enriched). The analysis yielded several significant findings:

Firstly, a highly significant main effect of enrichment was observed on CO_2_ consumption efficiency [F(1, 14) = 275.84, p < 0.000001]. This confirms that the enrichment procedure dramatically improved the cultures’ CO_2_ consumption capacity. Non-enriched bacterial cultures demonstrated a significantly lower CO_2_ consumption capacity when directly subjected to gas fermentation (mean CO_2_ consumption rates of 17.4% for AC, 21.2% for CF, and 21.4% for RF). In contrast, the progressive CO_2_ consumption rates observed during the 10-day cycle in enriched cultures of AC, CF, and RF were substantially higher, at 54.8%, 63.2%, and 82.1%, respectively (Fig. 4). This enhanced CO_2_ reduction in the enriched cultures is likely due to the selective enrichment and pretreatment of bacteria capable of acetogenic metabolism t, thus eliminating specifically suppressed methanogenic archaea and non-sporulating bacteria.

Secondly, a statistically significant main effect of culture type was also found on CO_2_ consumption efficiency [F(2, 14) = 10.41, p = 0.0017]. This indicates that, even when accounting for enrichment, the inherent capabilities of the different culture types (AC, CF, RF) to consume CO_2_ varied significantly. A progressive increment in gas consumption was noticed for all the reactors with an increase in cycle operation, attributed to bacterial adaptability and growth progression in response to the headspace gas mixture of CO_2_ and H_2_.

The reducing equivalent/electron donor, H_2_ gas, donates electrons to the CO_2_ fixation process, resulting in acetate as the primary product^26,28^. A successful gas reactor was set up for the enriched culture with the purging of the three gases CO_2_:H_2_:O_2_ and was run for three cycles. The CO_2_ reduction efficiency was higher in enriched bacterial cultures with a maximum CO_2_ consumption of 49.4% as compared to the non-enriched bacteria with a maximum of 17.6% (Fig. 4). The biomass accumulation was 0.455 g/L with a PHB content of 3.2%. As a preliminary stage, this study was triplicated, and the measurements were taken on average. CO_2_/H_2_ is taken up by the bacterial cell as bicarbonate ions or formyl tetrahydro folate (THF), which is then transformed into methyl THF, which is further converted into acetyl-COA, which is then taken up to produce PHB in the presence of PHA a, b, and c enzymes.

### Volatile fatty acids production

The cultures were evaluated for CO_2_ to VFA conversion following enrichment. Followed by RF and CF, the blend culture produced higher VFA of 3.6 g/L. The AC bioreactor generated 1.9–2 g/L VFA after 3 generations of enrichment. VFA production from pretreated MMCs from CF and RF is 2.3 and 3.1 g/L, respectively. VFA was 3.2 g/L in blend cultures. AC, RF, CF, and Blend had mean VFA concentrations of 2.017, 2.307, 3.243, and 3.467 g/L, respectively (Supplemental Table 1). VFA production significantly increased in pretreated cultures, as validated by a one-way ANOVA (F(3, 8) = 70.72, p < 0.001). Since acetogens have a lower affinity for hydrogen than methanogenic bacteria, this study’s main hypothesis was that inhibiting methanogenesis in the culture would increase the amount of hydrogen available for enhanced VFA synthesis. The production of VFA was an indication that the microbes present in the reactor have been enriched to take up a substrate and convert it into a product. The untreated inoculum culture yielded comparatively low VFA as most of the carbon was lost as methane gas, such as 890.7 mg/L, 1128 mg/L, and 1462.1 mg/L in untreated AC, CF, and RF, respectively. This depicts the metabolic efficiency of enriched bacteria in utilizing CO_2_ as substrate and H_2_ as electron donor driving the pathway towards acid synthesis (C_2_). The accumulation of acids could also lead to chain elongation. 

### PHB production

**Fig. 5:**
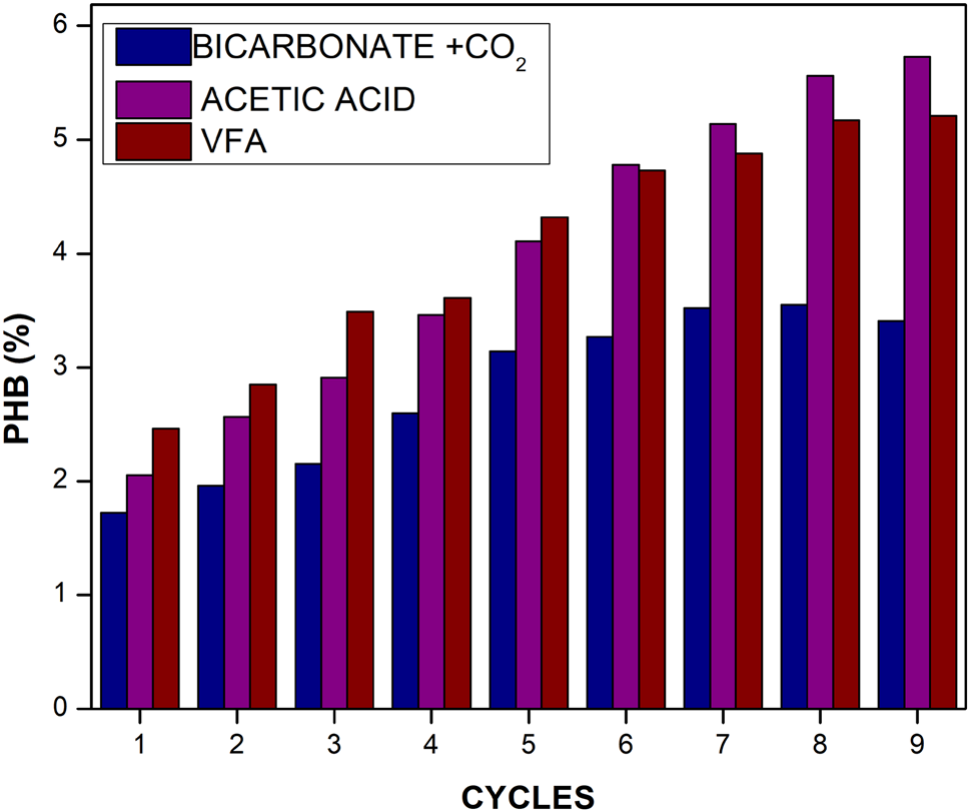
The comparative plot of CO_2_ reduction efficiency pattern by enriched and non-enriched MMC for PHB production.

PHB was attempted to produce with different substrates. The enriched culture VFA-PHB and CO_2_-PHB were fed with produced VFA and purged with a mixture of gases every 72 h respectively, and analysed for CO_2_ reduction, biomass accumulation and PHB content. The PHB production was observed to increase with each cycle, inferring the culture’s acclimation to the production of PHB. After enrichment, the total dry cell weight (DCW) of biomass obtained from bioreactors using sodium acetate, sodium bicarbonate, and CO_2_ gas as carbon sources was 4.45, 4.42, and 2.55 g/L, respectively. Correspondingly, the total PHB accumulation in these reactors reached 4.1% (0.182 g/L), 5.6% (0.236 g/L), and 3.2% (0.082 g/L), respectively. The PHB accumulation was 6.5% for enriched and 0.8% for non-enriched VFA-PHB cultures. The CO_2_ reduction and PHB accumulation was observed to be 49.9% and 5.02%, respectively for enriched cultures and 17.6% and 0.2% respectively for non enriched cultures. A one-way ANOVA was conducted to compare PHB production between these two carbon sources. The analysis revealed a statistically significance in PHB accumulation (F(1, 4) = 37.11, p = 0.0037) between cultures supplied with VFA as the carbon source and those supplied with CO_2_. The study infers that the organic acid derived from CO_2_ is an effective substrate for producing PHB (see supplementary Table 2) (Fig. 5).

The accumulated PHB is directly proportional to the number of cells, and a high cell density is desired to improve PHB production further with high biomass growth^29^. PHB production also depends on the diffusion capabilities of VFA and CO_2_ into the cells, where the transfer coefficient of CO_2_ is much lower than the organic acids^30^, hence proportional to the available carbon for product formation. The buffering capacity of the MMC to balance the pH between 6.5 and 7.5 also supports the PHB production. In some cases, the oxygen limitation will restrict growth and cause the undesired, early induction of the PHB accumulation phase, as described by Garcia-Gonzalez et al.^31^. Aiming to increase PHB production, the premature shift to PHB accumulation is caused by oxygen limitation. Studies also reported that a higher biomass concentration may be reached by prolonging the exponential growth phase, which can be achieved through a higher oxygen transfer rate (OTR). To enhance the yield the repeated fed –batch strategy was analysed as the approach was also suggested by Vlaeminck et al.^32^.

### Product analysis

**Fig. 6:**
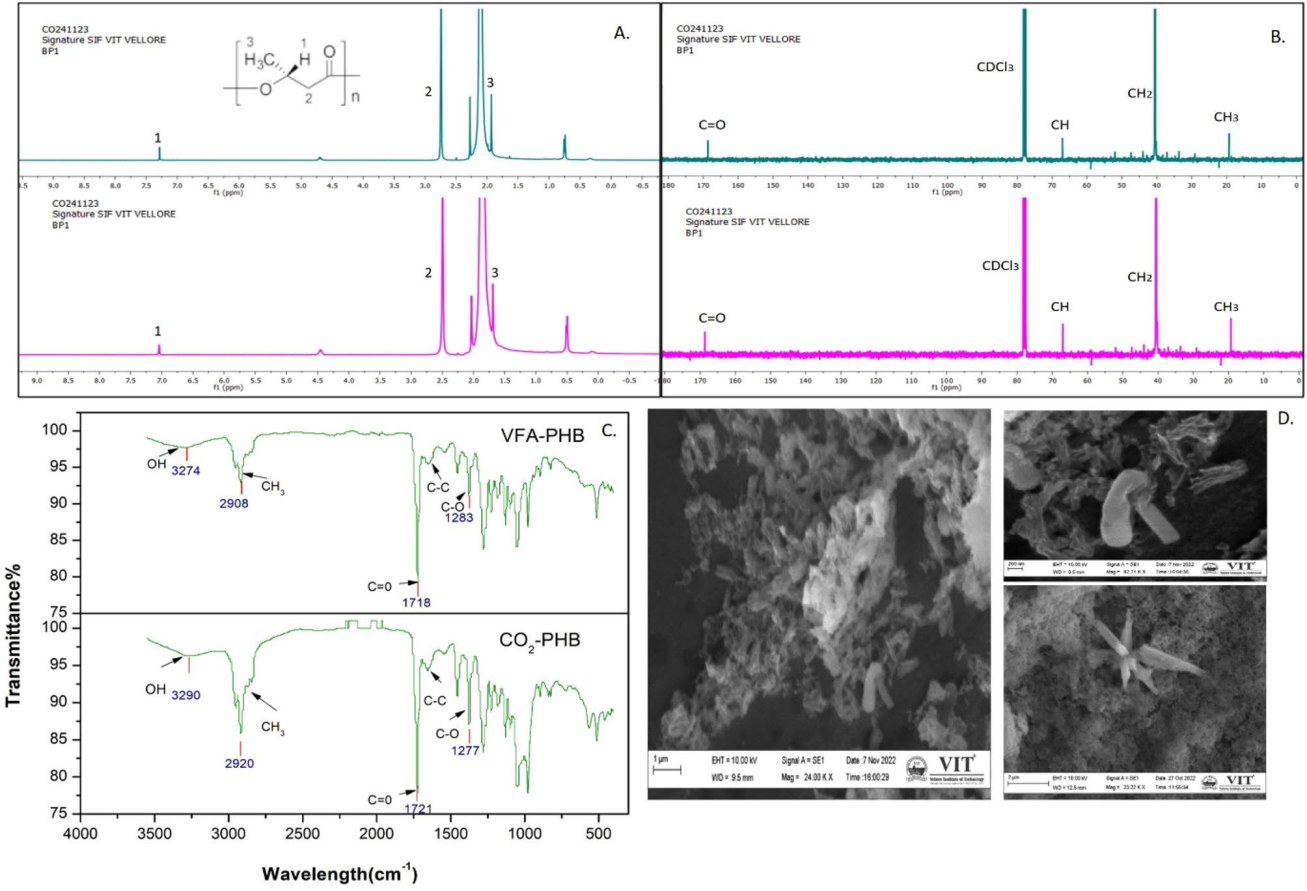
(**a, b**) H and 13C NMR Analysis for PHB molecule confirmation, (**c**) FTIR analysis for PHB molecule confirmation, for VFA-PHB and CO_2_-PHB; (**d**) SEM photograph of the mixed culture growing in glucose (top) and bicarbonate (bottom).

The SEM gives magnified image to study the size, shape, composition, crystallography, and synthesis of Exopolysaacchrides (EPS) developed in biomass between enriched and unenriched biocatalysts . The SEM images differentiate glucose- and bicarbonate-grown biocatalysts. However the unenriched culture consist more mucous content since the substrate was Glucose. However, mucous composition was much less in enriched culture because CO_2_ might make it chemoautotrophic. The enriched culture had a more effective mechanism for cell attachment and adhesion, according to SEM examination. The CO_2_-enriched MMC showed a decrease in the length and smaller bacteria than the unenriched, as reported by Tang et al.^33^. This morphology change could also be observed due to the organic to inorganic carbon shift. The cells under SEM were observed to have a rod-shaped structure for glucose-grown culture and a star-shaped morphology for bicarbonate-enriched culture. The star-shaped microbial community could be gram-negative *Alphaproteobacterium* sp., which is reported to be prominent in polluted water and sewage plants^34^. Hence, in the study the visual difference in the SEM analysis confirms the shift like biocatalysts towards the uptaking of CO_2_ (Fig. 6d).

The PHA’s FTIR spectrum revealed firm peaks at several wavelengths indicative of PHB. While the high peaks at 3000 and 2850 cm^−1^ are caused by the C–H stretching of alkanes, the solid and broad peaks at 3274 and 3290 cm^−1^ reveal the presence of O–H stretching of alcohol. Peaks at 1718/1721 cm^−1^ and a string of sharp peaks at 1283/1277 cm^−1^, respectively, could both corroborate the presence of the C=O and C–O stretch of ester. The small intermediary stretches are the C–C bonds between 1715 and 1300 cm^−1^ as seen in Fig. 6c^35,36^.

^1^H NMR have been used to examine the structural characteristics of the PHB generated. Three signals representing the doublets of the methyl group (CH_3_) and two multiples of the methylene group (CH_2_) and methine (CH) groups were found in the current investigation. These signals fall between 2 and 3 ppm, which is characteristic of the PHB polymer (Fig. 6a,b)^36^. The significant advantage of ^13^C NMR over ^1^H NMR is that, although proton NMR provides indirect information about the molecule’s carbon skeleton, ^13^C NMR provides direct information because, if the carbons are not equivalent, each molecule’s carbon provides a signal indicating whether or not hydrogen is linked to it. Organic molecules often have ^13^C signals between 0 and 220 ppm. The alkynes form between 70 and 80 ppm, while the aliphatic chemicals create a peak between 10 and 40 ppm.

### Carbon balance

**Fig. 7:**
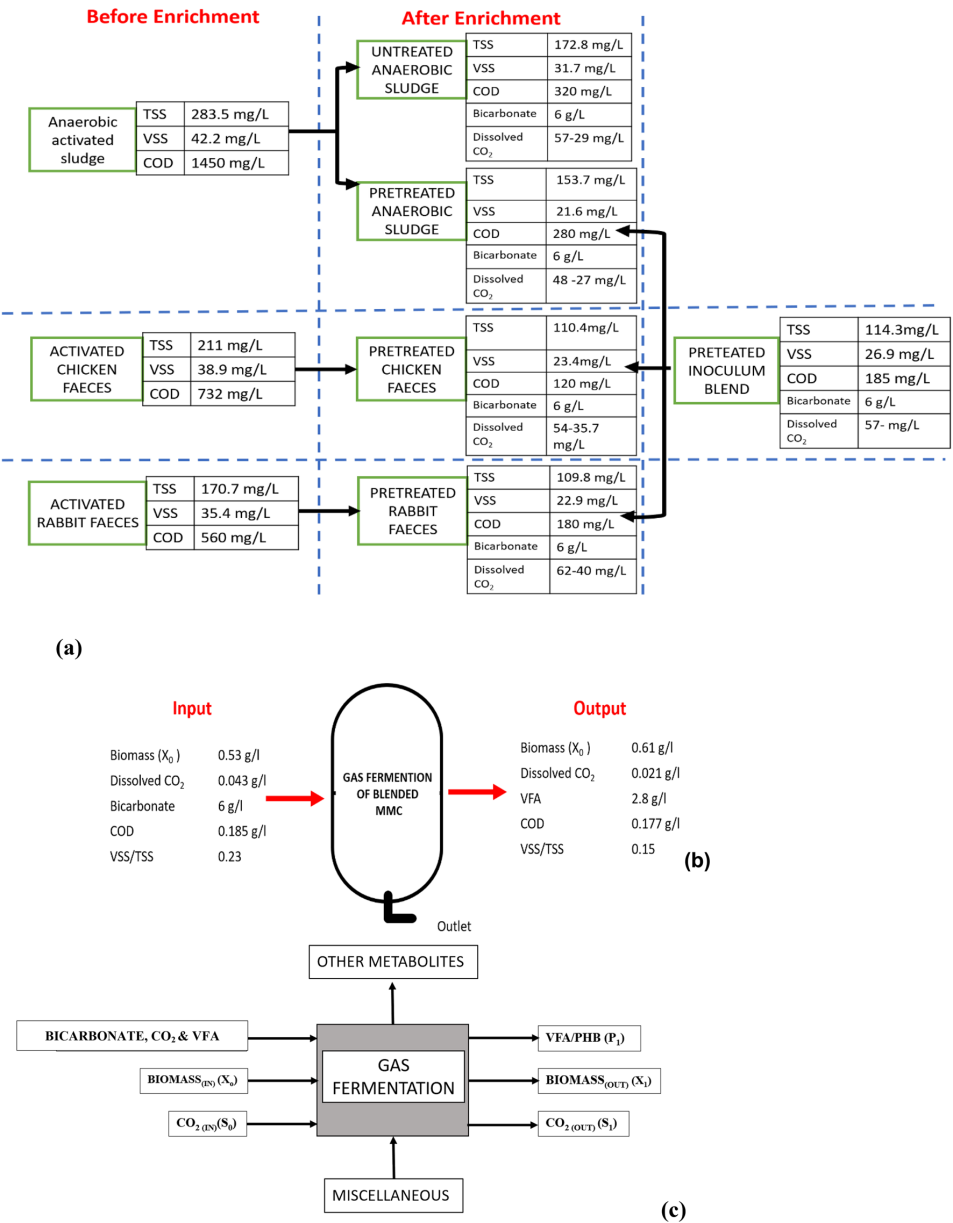
Mass balance for the PHB formation from various substrates.

Gas-to-liquid mass transfer of gaseous feedstock to value-added products in aerobic and anaerobic fermentation is difficult; understanding the carbon balance helps improve product output. Mass balance shows consortia enrichment for CO_2_ and bicarbonate absorption as feedstock. Organic biomass exhibits higher COD before enrichment, while COD decreases after enrichment, indicating the culture adapts to CO_2_ uptake. Figure 7 shows that organic and inorganic substrates become VFA and other residues. CO_2_ (C1) is dissolved in fluids and fixed by microbes into metabolites like short-chain fatty acids. After enrichment, medium, biomass, VFA, and PHB carbon concentrations were computed to monitor the carbon route (Fig. 7). The enrichment samples showed that fermentation transformed 12–20% of the original VSS into soluble COD. The primary success of the acidogenic process is the conversion of biodegradable COD to VFA^37^. The net fermentable yield (Y) of VFA in terms of COD was calculated as eq. (1)^38^,

1 $$Y=\frac{\Delta COD}{VSS}$$

Therefore, the fermentable yield of VFA from AC is 0.054 g COD/g VSS, similarly 0.026 g COD/g VSS for pretreated CF and 0.016 g COD/g VSS for pretreated RF and blend of all the mixed consortia is 0.06 g COD/g VSS.

**Fig. 8:**
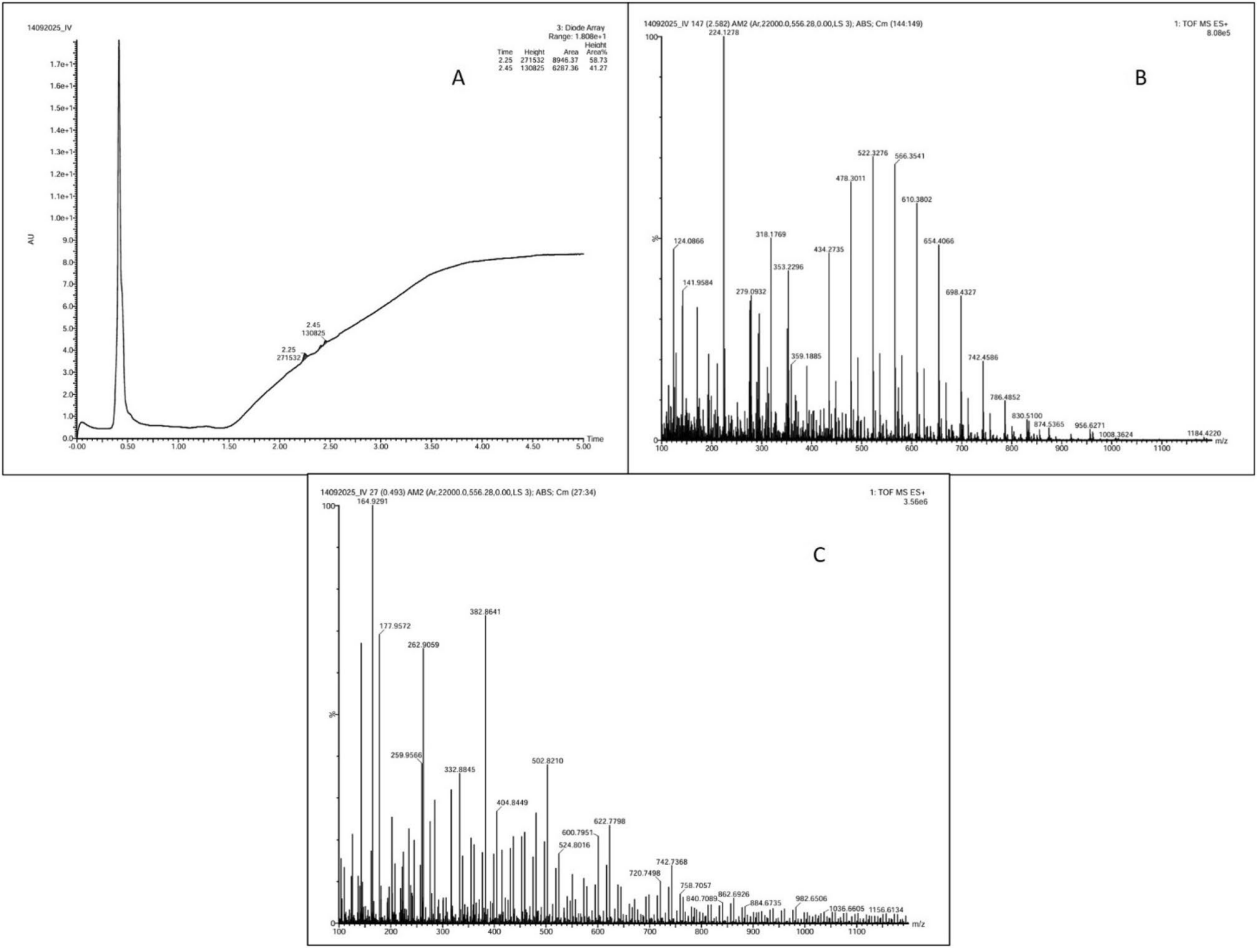
*LC-HRMS analysis of VFA-derived metabolites.* (A) LC chromatogram showing the detection of target analytes at a retention time of approximately 2.45 min. (B) Representative high-resolution mass spectrum (MS^1) displaying multiple ion peaks (m/z ≈ 141–1184) corresponding to diverse metabolites and possible adducts. (C) MS/MS fragmentation spectrum highlighting the diagnostic ions for metabolite identification and structural confirmation.

The experiment was performed in a closed system with a pulse feeding technique in the lab-scale reactors. To enhance the CO_2_ uptake capability of the inoculum culture, the headspace was filled with CO_2_ and H_2_ gas (20:80 v/v). The samples were collected every 24 h for the analysis of dissolved CO_2_. The density of CO_2_ gas (in g/L) at 2 atm pressures and 298 K temperatures is 3.58 g/L and was calculated using the Eq. (3):

2 $$PMw=dRT$$

where P is the total pressure, Mw is the molecular weight of the gas, d is density, R is the gas constant and T is the temperature. The volume of CO_2_ gas was calculated using Eq. (5).

3 $$Q=V*t$$

where Q is flow rate, V is volume, and t is time (2 min). Thus, by the mentioned condition, the input CO_2_ gas was sparged at a rate of 40 ml/min, so the volume of CO_2_ input is calculated to be 20 ml. It was found that 75% of the CO_2_ input was dissolved in the liquid-to-gas phase. Therefore, the dissolved CO_2_ level was considered while calculating the carbon mass balance. Once the stoichiometric chemical formula is known, trailing the carbon theoretically becomes more favourable. The raw chemical formula is C_a_H_b_O_c_N_d,_ where a, b, c, and d are the number of atoms present^38^. The carbon content of biomass at the start of the cycle was measured in terms of carbon percentage in cell dry weight as 200 ml of the previously enriched culture was transferred to the new medium. The input of carbon content as bacterial biomass for AC is 0.45 gC/L; CF is 0.4 gC/L; and RF are 0.34 gC/L. Similarly, the carbon mass percentage in sodium bicarbonate (input 6 g/L) was 14.3% with an input of 0.858 gC/L of carbon content. Input CO_2_ content was measured to be 48, 54, and 62 mg/L for the reactors containing AC, CF and RF, respectively. To evaluate the carbon content as output with the HRT of 3 days, Acetic acid and butyric acid were identified as the major volatile fatty acids (VFAs) with a 1.5:1 ratio (Based on typical retention time), which was confirmed by LC-HRMS analysis. The characteristic m/z peaks corresponding to acetic acid (m/z 61.03 [M–H]⁻) and butyric acid (m/z 87.04 [M–H]⁻) were observed, validating their dominance in the fermentation broth (Fig. 8). Additional peaks corresponding to propionic acid (m/z 73.03) and lactic acid (m/z 89.02) were also detected, suggesting minor carbon flux toward these intermediates. The carbon content in acetic acid is 40% and 54% in butyric acid. Hence the carbon content in acetic acid was 0.48, 0.6, 0.73 and 0.82 gC/L and butyric acid was 0.324, 0.41, 0.45 and 0.56 gC/L in AC, CF and RF respectively. The average increase in the carbon content in biomass during the enrichment process is approximately 20% for pretreated cultures and approximately 16% of biomass carbon content for untreated biomass. The carbon molecules may also have been fixed by other metabolites required for the maintenance and growth of the cell. The carbon content of biomass was determined to be 2.25, 2.13, and 1.29 gC/L, whereas the carbon content associated with PHB was 0.102, 0.132, and 0.046 gC/L, respectively, in sodium acetate-, sodium bicarbonate-, and CO_2_-fed bioreactors. These values indicate efficient CO_2_ fixation and subsequent conversion to both cellular biomass and polymeric carbon forms. The output values for carbon content in biomass is 0.3455 gC/L (approximately 13% increase), PHB is 0.22 gC/L (which corresponds to 0.39 g/L PHB polymer) and other metabolites are 0.565 g/L. The remaining dissolved CO_2_ at the end of the cycle is 25 mg/L. The carbon mass balance conformed to the enrichment of the mixed consortia for producing VFA and PHB with the uptake of CO_2_ as a carbon source.

This study illustrates the carbon balance and conversion efficiency at a laboratory scale utilising 500 mL serum bottles. However, it is important to recognize the difficulties involved in scaling this process to industrial reactors.

#### Thermodynamic studies for CO_2_ conversion to PHB

The standard Gibbs free energy of formation for relevant compounds, such as CO_2_, H_2_, acetate, and PHB, was used to calculate the thermodynamic efficiency of CO_2_ reduction to PHB. The following equations were used to calculate the Gibbs free energy for the reactions:

$$2{CO}_{2 }+4{H}_{2}\to {CH}_{3}CO{O}^{- }+2{H}_{2}O$$

$$2{CH}_{3}CO{O}^{- }+2 {O}_{2} \to \left({C}_{2}{H}_{4}{O}_{2}\right)n+2C{O}_{2}$$

where, ΔG°f for CO_2_, H_2_, 3-HB and acetate was taken from standard thermodynamic databases, the conversion of acetate to PHB was assumed to be near stoichiometric, with PHB accumulation proportional to biomass growth.

### Thermodynamic efficiency of CO_2_ reduction

The calculated Gibbs Free Energy Change (ΔG) for the reduction of CO_2_ to acetate and subsequently PHB production was found to be negative, indicating that the reactions are thermodynamically favorable.

The ΔG for CO_2_ reduction to acetate

4 $$2{CO}_{2 }+4{H}_{2}\to {CH}_{3}CO{O}^{- }+2{H}_{2}O$$

Gibbs free energy of acetate formation under STP (298 K, 1 atm) was taken to be − 370.3 kJ mol^−1^^39^, for CO_2_ − 394 kJ mol^-1^ and for H_2_ as 0^40^. Gibbs free energy change under standard conditions (298 k, 1 atm) is calculated using Eq. (5

5 $$\Delta G^\circ = \sum \Delta {G}_{f}^\circ \left(product\right)-\sum \Delta {G}_{f}^\circ (reactant)$$

Substitute Gibbs free energies of formation for CO_2_ and acetate Eq. (6):

6 $$\Delta G^\circ =\left[\Delta {G}_{f}^\circ \left(acetate\right)\right]-[\Delta {G}_{f}^\circ \left(C{O}_{2}\right)+4*\Delta {G}_{f}^\circ \left({H}_{2}\right)]$$

The $\Delta G^\circ$ value was calculated to be 23.7 kJ/mol at standard conditions. This positive value confirms that the CO_2_ reduction is non spontaneous. This non-spontaneous process highlights how bacteria can harness energy to convert inorganic CO_2_ into usable organic carbon (acetate), which can then be stored or used in energy-intensive biosynthetic pathways like PHB production.

For PHB production from acetate:

Similarly, Gibbs free energy of 3-HB formation under STP (298 K, 1 atm) was taken as − 361.84 kJ/mol^41^ and for H_2_O is − 237.13 kJ/mol. Substitute Gibbs free energies of formation for 3-hydroxybutyrate, H_2_O and acetate in Eq. (7):

7 $$\Delta G^\circ =\left[\Delta {G}_{f}^\circ \left(3-HB\right)+\Delta {G}_{f}^\circ \left({H}_{2}O\right)\right]-\left[2* \Delta {G}_{f}^\circ (acetate)\right]$$

∆G° was computed as 141.63 kJ/mol under normal circumstances. To form polyhydroxybutyrate (PHB) from acetate, a positive Gibbs free energy (ΔG > 0) indicates a non-spontaneous process that requires external energy input, such as ATP hydrolysis or reducing equivalents in enzymatic pathways. That cells prefer PHB production only when energy and carbon are abundant suggests that it is tightly regulated. In nutrient-limited or energy-scarce settings, the pathway may be downregulated to prioritise more immediate metabolic processes, making PHB synthesis a controlled, energy-intensive carbon and energy storage approach. The positive Gibbs free energy (ΔG > 0) for polyhydroxybutyrate (PHB) synthesis from acetate suggests a thermodynamically unfavourable process.

Polymerisation reduces disorder and takes a lot of energy, such as ATP or reducing equivalents. However, this negative reaction helps the organism strategically. PHB stores carbon in nitrogen- and phosphorus-poor environments. Cells expend energy to create PHB as an energy store, making this mechanism physiologically necessary yet energetically costly under certain environmental conditions.

In this part, biochemical reactions’ thermodynamic favorability and energy requirements are explained to comprehend their potential. Detailed energy balancing, including CO_2_ compression and gas sparging, improves techno-economic evaluation, improving industrial feasibility and sustainability.

### KEGG and 16S rRNA analysis for PHB producing MMC

**Fig. 9:**
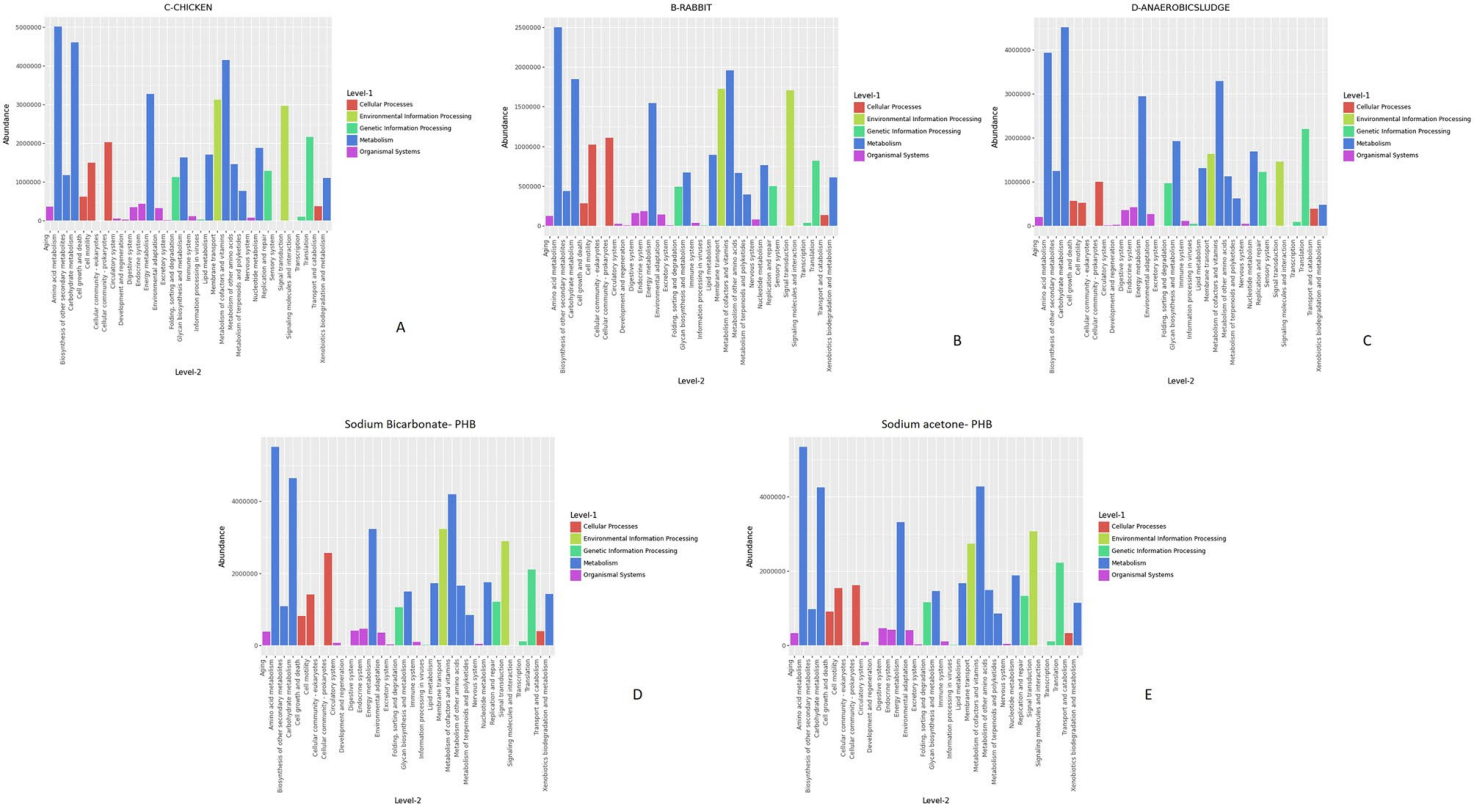
(**A**–**C**) Functional analysis of VFA producing enriched mixed microbial culture; (D&E) Functional analysis of PHB producing enriched mixed microbial culture.

**Fig. 10:**
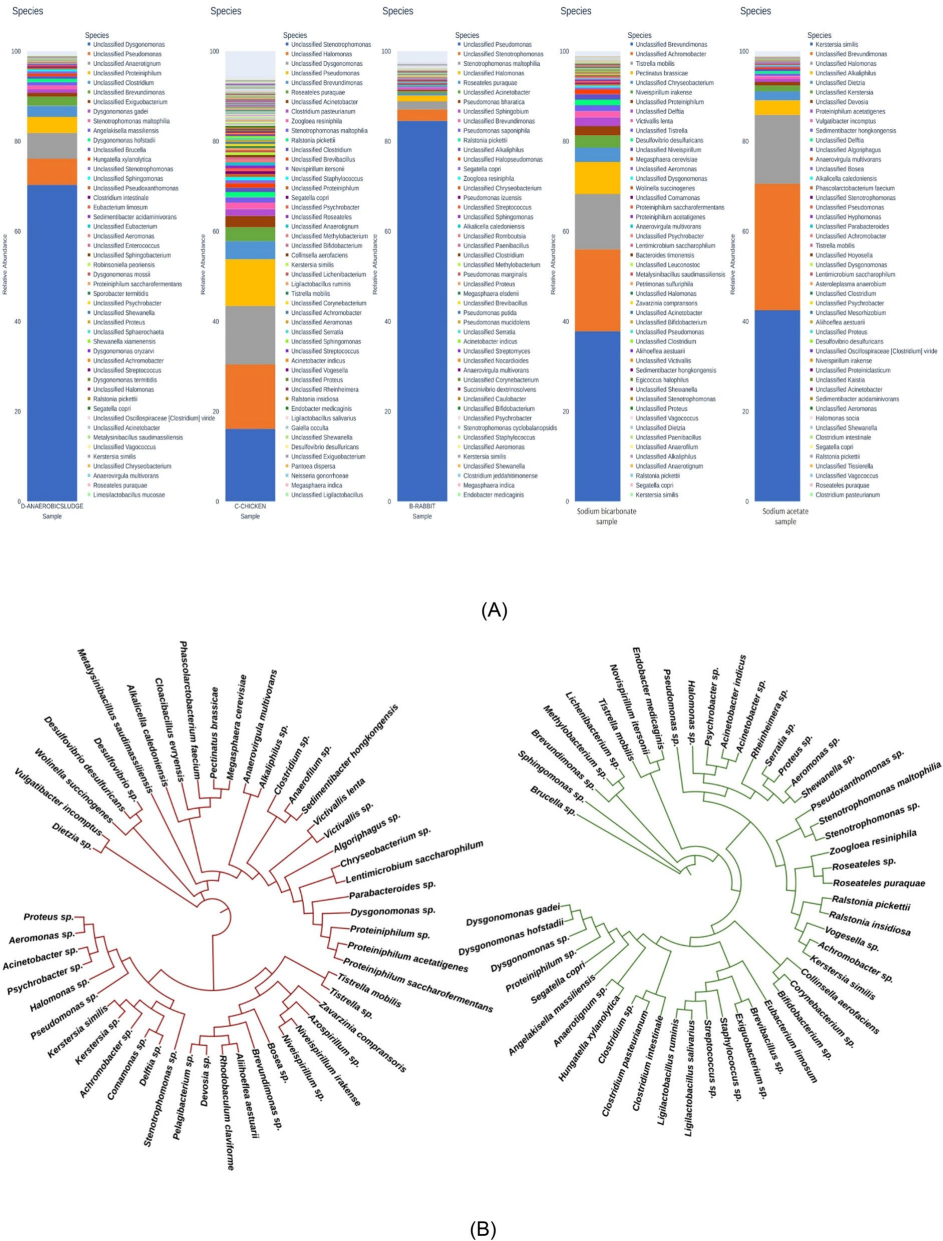
(**A**,**C**) metagenoic analysis of VFA producing enriched mixed microbial culture respectively; (**B**,**D**) phylogenic tree of PHB producing enriched mixed microbial culture, repectively.

The dicarboxylate/4-hydroxybutyrate and 3-hydroxypropionate cycles can aerobically fix CO_2_^42^. PHB production relies entirely on fermentation conditions; oxygen concentration, feeding strategy, and nutrient restriction . In this enrichment process, feeding techniques like pulse or aerobic dynamic feeding were used, where the feed was supplemented every 72 h based on the observation of biomass growth (at OD_600_)^43,44^. Nitrogen and phosphorus deficit conditions cease protein and nucleic acid synthesis, leading to high amounts of NADH and NADPH accumulation. As a result, citrate synthase and isocitrate dehydrogenase are inhibited, causing the TCA cycle to slow down and acetyl-CoA to be channeled toward PHB production. The precursor for PHB is 2 molecules of acetyl-CoA, and its conversion process employs 3 enzymes, PHA a,b, and c, which are controlled by a single operon^45,46^. AC contains a variety of microorganisms, mainly *Unclassified Dysgonomonas* and *Pseudomonas*, along with some *Unclassified Clostridium* and *Brevundimonas*. This composition suggests anaerobic fermentation or digestion. In contrast, *unclassified Pseudomonas* and *Stenotrophomonas* dominate the rabbit sample (Fig. 10A). A less diversified but perhaps highly specialised microbial community is indicated. In the CF (Fig. 9A), *unclassified Stenotrophomonas*, *Dysgonomonas*, and *Pseudomonas* dominate. Other genera like *Unclassified Methylobacillus* and *Sphingomonas* are more widespread, suggesting more diversity than the rabbit sample. The diagrams’ KEGG pathway analysis (Level 1 & 2) supports CO_2_ assimilation for VFA generation in acetogenic bacteria (Fig. 9A–C).

High abundance in metabolism pathways (blue bars in Level 1) indicates CO_2_ assimilation for VFA synthesis. Under 'Metabolism,' Level 2 subcategories like "Carbon fixation pathways in prokaryotes" indicate CO_2_ assimilation potential, energy metabolism pathways related to energy generation since CO_2_ fixation is energy-intensive, and carbohydrate metabolism pathways that use carbon sources. Acetogenic bacteria are abundant in “metabolism” pathways, especially those associated with acetate production (pyruvate metabolism, glycolysis/gluconeogenesis to acetyl-CoA). Acetogen-related processes like the WLP under "Carbon fixation pathways" or "Metabolism of cofactors and vitamins."

Analysis of the microbial consortia revealed distinct community structures contingent upon the primary carbon substrate. In the culture supplied with sodium bicarbonate, the dominant genera included *Brevundimonas, Achromobacter, Tistrella mobilis*, and *Pectinatus brassicae* (left) (Fig. 10A). The prevalence of these taxa under conditions favoring inorganic carbon uptake suggests their role in the assimilation of CO_2_ for subsequent PHB synthesis. Conversely, the consortium enriched with sodium acetate was significantly dominated by *Kersersia similis, Brevundimonas*, and *Halomonas* (right) (Fig. 10A)*.* The abundance of these organisms is consistent with an established metabolic capacity to utilize acetate, a direct precursor, for efficient PHB accumulation.

Metagenomic analysis and KEGG pathway mapping substantiated the functional potential of the microbial communities for PHB production (Fig. 9D,E)^47–49^. A high abundance of genes associated with carbohydrate, amino acid, and energy metabolism was identified, indicating a robust capacity for generating acetyl-CoA, the central intermediate for PHB biosynthesis. The genetic framework for carbon fixation was supported by the presence of key species, including *Tistrella mobilis,* and acetogenic bacteria such as *Prosthecochloris* and *Anaerovirgula multivorans*. Furthermore, the high representation of genes related to genetic information processing is indicative of metabolically active populations, confirming that the consortia harbor the necessary microbial taxa and functional genes for efficient CO_2_ assimilation and bioplastic production.

While a phylogenetic tree derived from 16S rRNA gene sequences cannot directly predict functional traits, it permits inferences based on evolutionary relatedness (Fig. 10B). The capability for PHB production is not encoded by the 16S rRNA gene, yet phylogenetic proximity to known PHB-producing taxa can suggest a shared metabolic potential. For instance, species that cluster closely with characterized PHB producers, such as members of the genera *Pseudomonas* or *Bacillus*, are more likely to possess this trait. In contrast, phylogenetically distant organisms are less likely to share such functional capabilities. Therefore, the phylogenetic analysis serves as a valuable correlative tool, complementing functional genomic data by identifying potential candidate organisms based on their taxonomic placement relative to characterized species.

## Discussion

PHB-producing bacteria that use organic and inorganic carbon fractions as substrates improve environmental and resource sustainability. Valuing waste streams turns a linear economy into a CE. PHB’s superior crystallinity and reduced oxygen permeability make it a possible option for petroleum-based polymers like PP and PE^50^, offering superior barrier permeability and thermoplasticity^51^. Its inherent biodegradability is a key factor enabling its role in a CE, distinguishing it from conventional plastics and aligning with Sustainable Development Goal (SDG) 12.

Recent research highlights the critical role of microbial communities and specific enzymes in the degradation of bioplastics, including PHB. The review article by Zhou et al. (2023), details their production from waste streams and complete biodegradation in environments like soil, marine, and compost. The overview discusses the enzymatic mechanisms, primarily involving PHA depolymerases, that break down the polymers. It elaborates on how degradation rates are influenced by the polymer’s properties, such as its chemical composition and crystallinity, and by environmental conditions like temperature and oxygen availability^52^. These insights reinforce PHB’s potential for sustainable waste management, contributing to SDG 13 and 15.

Methanogens, homoacetogens, acidogens, Actinobacteria, and both aerobic and anaerobic microbes contribute to CO_2_ conversion through multiple carbon fixation pathways. Within this closed-loop CE framework, the microbial consortium effectively valorizes CO_2_, enabling sustainable bioprocesses that align with the principles of circular bioeconomy and Sustainable Development Goal (SDG) 9. The pH values across experimental setups ranged between 5.5 and 7.2, showing distinct effects on carbon metabolism. Lower pH (5.5–6.2) promoted VFA accumulation by favoring acidogenic bacteria, while near-neutral pH (6.8–7.2) enhanced PHB synthesis by enabling stable acetogenic activity and balanced CO_2_ assimilation. These results highlight the crucial role of pH in steering metabolic pathways toward either intermediate (VFA) or storage (PHB) products, consistent with prior observations in CO_2_-fed mixed culture fermentations^18,21^.

Two primary valorization routes were identified in this study. First, a pretreatment followed by VFA-enriched cultivation facilitated the conversion of CO_2_ into volatile fatty acids (VFAs), which subsequently served as precursors for PHB synthesis. Second, PHB-enriched microbial cultures demonstrated the direct biosynthesis of PHB using CO_2_ as the carbon source. The resulting polymer can be efficiently extracted, characterized, and utilized; following its use, the biodegradable product can be composted and reintroduced as substrate for further cultivation, thereby completing the circular cycle of carbon utilization^42–44^. This integrated system—from CO_2_ fixation to biodegradable polymer formation and compost reintegration—enhances resource efficiency and supports SDG 6 by minimizing waste and promoting sustainable water and sanitation management.

At the enzymatic level, this work highlights the intrinsic connection between pathways involved in PHA synthesis and degradation, underscoring how microbial metabolism underpins the circular bioeconomy. The ability of these metabolic routes to recycle carbon from waste streams into new value-added products contributes to reduced plastic pollution and improved resource recovery, advancing SDGs 8 (Decent Work and Economic Growth) and 11 (Sustainable Cities and Communities)^53,54^. Furthermore, adaptive shifts in CO_2_ assimilation pathways allow for the biosynthesis of diverse bioproducts, reflecting the metabolic versatility and potential for developing various bio-based materials within this sustainable framework.

Comparative evaluation with recent studies^13,14,18^ highlights that our enriched MMC achieved PHB accumulation of 0.82 g·L⁻^1^ (3.2 ± 0.18% DCW) and a CO_2_/VFA conversion efficiency of more than 43.5%, which lies within the range reported for mixed microbial consortia (1.5–4.5 g·L⁻^1^; 25–45% DCW). In contrast, engineered *Cupriavidus necator* cultures and membrane-assisted systems have achieved higher PHB titers of 6–12 g·L⁻^1^ (60–80% DCW) with CO_2_ conversion efficiencies approaching 70–85%^62^. Despite these differences, our process demonstrates comparable carbon fixation potential under non-sterile conditions, without the need for genetic modification or membrane support. This establishes it as a feasible proof-of-concept rather than a fully optimized production platform. The observed increase in PHB accumulation may partly reflect stress-induced carbon storage or community-level shifts rather than inherently superior metabolic efficiency. Moreover, modest PHB yields, CO_2_ mass transfer limitations, and the need for improved reactor control remain key constraints to be addressed. Collectively, these insights offer a balanced and statistically grounded perspective on the feasibility and limitations of MMC-based CO_2_-to-PHB bioconversion.”

Overall, this study demonstrates that converting CO_2_ into PHB and VFAs through microbial valorization significantly enhances the sustainability of the Carbon Bioeconomy . The developed bioprocess integrates CO_2_ sequestration with biopolymer and organic acid production, thereby contributing to a low-carbon and resource-efficient industrial model. The five-generation enrichment strategy improved microbial carbon fixation capacity and CO_2_ consumption, leading to elevated PHB accumulation. Metagenomic insights confirmed enhanced metabolic potential within the enriched consortia, supporting sequential pathways for VFA and PHB biosynthesis. Collectively, this research establishes a scalable and environmentally responsible route for transforming waste CO_2_ into biodegradable polymers and valuable intermediates, reducing dependency on fossil-derived plastics and promoting global climate resilience in line with SDGs 6, 9, 12, and 13.

## Material and methods

### Experimental overview

The experiments were performed in two phases of operation. In phase I, the enriched cultures were evaluated for VFA production using CO_2_/bicarbonate as substrate and PHB production using CO_2_/bicarbonate and sodium acetate as carbon sources. In phase II, VFA produced from the phase I operation was recirculated as a substrate for the production of PHB. Further comparative analysis of CO_2_ to PHB and VFA to PHB was studied.

### Collection of mixed microbial culture (MMC)

Three indegenous samples of MMCs were collected from a wastewater treatment plant-WTP (VIT, Vellore) and local animal farm (in Katpadi, Vellore) as anaerobic sludge (AC), Rabbit faeces (RF) and Chicken faeces (CF) for VFA production. The bacterial cultures AC, RF and CF were activated by adding 6 g/L of glucose every 24 h for 5 days with continuous stirring at 150 rpm in anaerobic condition until the maximum biomass growth was obtained. The three anaerobic cultures were individually heat treated to 80 ± 5 °C for 2 h, followed by acid treatment with orthophosphoric acid (pH 3) for 24 h to suppress the methanogenic bacterial population^23^. Similarly, distinct MMC’s collected from an aerobic wastetank of WTP (VIT, Vellore) for PHB production was operated in aerobic condition and was activated with glucose. A drastic reduction in pH and high biomass growth was observed after activation (see supplementary Fig. 2).

Further, the physical parameters such as pH, ORP, and OD_600_ were measured using Systronics Digital pH meter335 and HITACHI UH5300 spectrophotometer. These activated cultures were preserved in an airtight container at 4 °C and were used as required. To enrich all the cultures, a simulated growth media i.e. designed synthetic wastewater was prepared with a composition of NH_4_Cl (0.5 g/L), KH_2_PO_4_ (0.25 g/L), K_2_HPO_4_ (0.25 g/L), MgCl_2_.6H_2_O (0.3 g/L), FeCl_3_ (0.025 g/L), NiSO_4_ (0.016 g/L), CoCl_2_ (0.025 g/L), ZnCl_2_ (0.015 g/L), CuCl_2_ (0.0105 g/L), CaCl_2_ (0.005 g/L) and MnCl_2_ (0.015 g/L^23^. No pretreatment was done for the aerobic MMC sample (see supplementary Fig. 1).

### Bioreactor and selective enrichment process

Enrichment of homoacetogenic autotrophs was conducted in the batch mode by growing the bacteria in 1 L serum bottles with rubber sealed alluminium heads and an inlet valve that could withstand 2 Barr pressure, with a working volume of 500 ml (2(microbial culture):3(media composition)). The five generations of enrichments (G0, G1, G2, G3, G4) were performed subsequently, with each generation performing three cycles (triplicates) and an HRT of 72 h. At G0 (6:0), the cultures were added with 6 g/L of glucose as a sole carbon source also served as activation; at G1 (2: 4) 2 g/L of bicarbonate along with 4 g/L of glucose, at G2 (4:2) 4 g/L of bicarbonate along with 2 g/L of glucose and at G3 (0:6) added with 6 g/L of bicarbonate as the carbon alternative followed by G4 sparging of CO_2_:H_2_ gas at 20:80 (v/v) ratios (Table.2). At the end of the enrichment process, the samples were collected for VFA analysis, bicarbonate reduction and CO_2_ consumption^21^. After the enrichment process of G3, 100 ml of each culture was used as inoculum in the blended form to study the combined VFA production and CO_2_ reduction. For the fermentation of CO_2_, reactors were operated at room temperature and to ensure uniform dispersion, a magnetic stirrer operating at 120 rpm was used in the reactors to permit mixing.

**Table 2:** Sequential enrichment generations.

Generations	Glucose	Alternate carbon source	Mixture of gases	HRT (hrs)	No. of cycles	Days of operation
G0	6	0	0	72	3	9
G1	4	2	0	72	3	9
G2	2	4	0	72	3	9
G3	0	6	0	72	3	9
G4	0	6	CO_2_:H_2_/CO_2_:H_2_:O_2_	72	3	9

The bacterial screening in aerobic MMC’s was performed where the enriched cultures were serially diluted, plated, and stained with Sudan Black B, thus confirming the lipid-filled opaque inclusions inside the bacterial cell where two distinct carbon sources were supplied to Generations 1- 3 enrichments (same as above; Table 2) for every 72 h. The sodium acetate was used as a carbon source during enrichment to facilitate the uptake of VFA (majorly acetic acid)^55^ and sodium bicarbonate was used as another carbon source to facilitate the metabolism of bicarbonate ions. Further, supplied with the CO_2_ to bicarbonate conversion and metabolism and the culture were denoted as CO_2_-PHB.

### Extraction of VFA and PHB

The VFA extraction was done with the initial one-step liquid–liquid extraction with the help of dichloromethane (DCM). The separating funnel was used to discard the water component and the DCM dissolved organic acids were extracted using a rotary evaporator (IKEA-RV8/CHILLER)^56^. PHB extraction was performed by following the ultrasonic and solvent-free extraction method, as mentioned by Martínez-Herrera et al.^57^. The biomass was collected and washed using centrifugation and the bacterial cell wall was broken down by subjecting it to ultrasound vibrations for 30 min in the presence of 5% (v/v) sodium hypochlorite solution. The PHB was extracted with the assistance of hot chloroform. After the PHB extraction, the percentage accumulation of PHB was done, where the cellular biomass was collected by centrifugation (REMI R-24 plus microcentrifuge) of 5 mL of growth media at 1900 g for 15 min to separate biomass from the fermentation broth, the ampules were pre-weighed as 2-mL microtubes. The biomass pellets were dried at 60 °C for 24 h using a hot air oven after being washed with distilled water. The dried biomass was weighed to calculate the dry cell weight (DCW). The PHB accumulation percentage (%PHB) was estimated as the PHB percent composition present in the cellular biomass using the following formula Eq (8):

8 $$\% PHB=\left(\frac{PHB(g)}{DCW (g)}\right)*100$$

### Instrumental analysis

The pH and oxidation–reduction potential (ORP) were measured using a *Systronics Digital pH meter 335*. Biomass growth was quantified at OD₆₀₀ (600 nm) using a *Hitachi UH5300 UV–Vis spectrophotometer*. Dissolved CO_2_ was monitored with a *Mettler Toledo CO*_2_ analyser (probe method), while bicarbonate concentration was determined titrimetrically against standard sulfuric acid using phenolphthalein and methyl orange as indicators. VFAs were examined by LC–HRMS (Liquid Chromatography–High Resolution Mass Spectrometry), where retention time (RT) and mass-to-charge (m/z) values were used to confirm the presence of individual VFAs. Enriched cultures were visualized using scanning electron microscopy (SEM) with *Thermo Fisher-FEI QUANTA 250 FEG* (VIT facility). Chemical oxygen demand (COD) was determined by the closed reflex method, while total suspended solids (TSS) and volatile suspended solids (VSS) were measured according to APHA IS-3025 protocol^14^. Polyhydroxybutyrate (PHB) was extracted using ultrasonic-assisted solvent-free extraction methods, and characterized by Fourier Transform Infrared Spectroscopy (FTIR) using a *Thermo Nicolet IS50 FTIR spectrometer with ATR module* (Thermo Fisher, VIT facility). Structural confirmation was further performed using ^1^H and ^13^C Nuclear Magnetic Resonance (NMR) spectroscopy on a *Bruker Avance III 400 MHz spectrometer* (VIT facility), with CDCl₃ as the solvent^35,36^.

### High-throughput sequencing and KEGG analysis

Microbial community analysis was conducted on samples from all the optimized cultures of PHB-producing bacteria. This entire process was outsourced to Biokart India Pvt. Ltd. (Bengaluru, Karnataka). Total genomic DNA was extracted from the collected samples. The V3-V4 hypervariable region of the 16S rRNA gene was then amplified using Phusion High-Fidelity DNA Polymerase. Specific primers used for this amplification were 16sF (5ʹ AGAGTTTGATGMTGGCTCAG3ʹ) and 16sR (5ʹ TTACCGCGGCMGCSGGCAC3ʹ). The primary PCR reaction comprised a 25 µl volume, containing 0.5 µM of each primer, 0.2 mM dNTPs, and 20 ng of template DNA. Thermal cycling involved an initial denaturation at 98 °C for 30 s, followed by 25 cycles of denaturation (98 °C for 10 s), annealing (55 °C for 30 s), and extension (72 °C for 30 s), with a final extension at 72 °C for 5 min.

A subsequent barcoding PCR was performed using Q5 Polymerase in a 20 µl reaction, including 0.4 µM primers, 0.1 mM dNTPs, and 5 µl of the first PCR product. This involved 8 cycles, with conditions similar to the primary PCR: 98 °C for 30 s; 8 cycles of 98 °C for 10 s, 60 °C for 30 s, 72 °C for 30 s; and a final extension at 72 °C for 2 min. Post-amplification, 16S rRNA gene products underwent purification and quality assessment via gel electrophoresis and spectrophotometry. Amplicons with a 260/280 nm absorbance ratio of 1.8–2.0 were deemed suitable for further analysis.

The purified amplicons were sequenced on the Illumina MiSeq Platform. Raw reads were demultiplexed and processed. Quality control utilized FastQC (v0.11.9) and MultiQC (v1.10.1), followed by stringent filtering to remove low-count and low-variance features. Chimera detection was a critical part of the bioinformatics workflow, ensuring robust Operational Taxonomic Unit (OTU) generation. OTU clustering and taxonomic assignment were performed against the NCBI database. Data normalization was achieved through rarefaction and TSS scaling, and microbial abundance was visualized using heatmaps.

For phylogenetic analysis, the identified OTU sequences were retrieved in FASTA format from GenBank. Multiple sequence alignment was conducted using MEGA 6.0 software. A phylogenetic tree was constructed using the Neighbor-Joining algorithm with 1000 bootstrap iterations, yielding a consensus tree in NEWICK format. This tree was then visualized and analyzed using the Interactive Tree Of Life (iTOL, http://itol.embl.de/) online software^58^. Functional insights into the microbial communities were predicted using PICRUSt2 for KEGG pathway analysis^59^.

### Mass balance

Mass balance calculations were conducted to compare the performance of untreated and pretreated cultures with that of enriched and unenriched cultures. Physical parameters pertaining to COD, total suspended solids (TSS), and volatile suspended solids (VSS) were examined for every sample to assist the products’ organic and inorganic carbon conversion analysis. In the gas fermenter, the carbon was evaluated in the form of dissolved CO_2_ gas (analysed by CO_2_ analyser), sodium bicarbonate (NaHCO_3_-6 g/L) and the inoculum (cell dry weight-VSS) Eq. (9). Chemical or gravimetric techniques are frequently used to characterise complex organic materials. Stoichiometry can conveniently retrieve the elemental makeup of an organic material. The molecular formula for biomass in VFA fermentation was taken to be C_60_H_87_N_12_O_23_P^60^.

9 $${\mathrm{C}}_{{{\mathrm{TOTAL}},{\mathrm{IN}}}} = {\text{ C}}_{{{\mathrm{CO2}},{\mathrm{IN}}}} + {\text{ C}}_{{{\mathrm{NAHCO3}}}} + {\text{ C}}_{{{\mathrm{BIOMASS}},{\mathrm{IN}}}} + {\text{ C}}_{{{\mathrm{MISCELLANEOUS}}}}$$

For carbon content at the end of HRT (3 days) were accounted for VFA (mainly acetic acid and butyric acid), the increased biomass, dissolved CO_2_ remaining (CO_2_, _OUT_) and other metabolites Eq. (10) (supplementary Table 5).

10 $${\mathrm{C}}_{{{\mathrm{TOTAL}},{\mathrm{IN}}}} = {\text{ C}}_{{{\mathrm{CO2}},{\mathrm{OUT}}}} + {\text{ C}}_{{{\mathrm{CH3COOH}}}} + {\text{ C}}_{{{\mathrm{C4H8}}0{2}}} + {\text{ C}}_{{{\mathrm{BIOMASS}},{\mathrm{OUT}}}} + {\text{ C}}_{{{\mathrm{METABOLITES}}}}$$

Similarly the carbon balance for PHB cell growth, i.e., biomass production and PHB accumulation was calculated with the molecular formula as C_4.09_ H_7.13_ O_1.89_ N_0.76_ and C_4_H_6_O_2_^61, respectively^. The following equation was followed Eq. (11) and carbon mass percentage was calculated to balance the input and output carbon for PHB production.

11 $${\mathrm{C}}_{{{\mathrm{TOTAL}},{\mathrm{IN}}}} = {\text{ C}}_{{{\mathrm{CO2}},{\mathrm{OUT}}}} + {\text{ C}}_{{{\mathrm{C4H6}}0{2}}} + {\text{ C}}_{{{\mathrm{BIOMASS}},{\mathrm{OUT}}}} + {\text{ C}}_{{{\mathrm{METABOLITES}}}}$$

## Supplementary Information

Supplementary Information.

## Data Availability

The datasets generated and analyzed during the current study are available in the NCBI Sequence Read Archive (SRA) repository under BioProject accession number PRJNA1302704. Individual sample accessions are listed in Supplementary Table S1.
