# Supplementary material for: Sustainable CO2 valorization for PHB production towards circular economy: metagenomic insights on enriched indigenous microbial cultures
Source: Sci Rep. 2026 Jan 10;16:1552. doi: 10.1038/s41598-025-26791-7 (PMC12799609; doi:10.1038/s41598-025-26791-7)
Supplement: Supplementary file 1 — Supplementary Information. [file 41598_2025_26791_MOESM1_ESM.docx]

**Sustainable CO₂ Valorization for PHB production towards Circular Economy: Metagenomic Insights on Enriched Indigenous Microbial Cultures**

**Isha Bodhe ^a,b^ and Velvizhi Gokuladoss ^b^***

*^a^ School of Biosciences and Technology, Vellore Institute of Technology, Vellore, India.*

*^b^ CO_2_ Research & Green Technologies Centre, Vellore Institute of Technology, Vellore, India.*

*(*Corresponding Author* Email: [velvizhi.g@vit.ac.in](mailto:velvizhi.g@vit.ac.in)*)*

**SUPPLEMENTARY INFORMATION**

**List of supplementary figures-**

**Figure 1: Enrichment process for PHB and VFA production.**

**Figure 2:Activation for MMC from chicken, sludge and rabbit sample.**

**
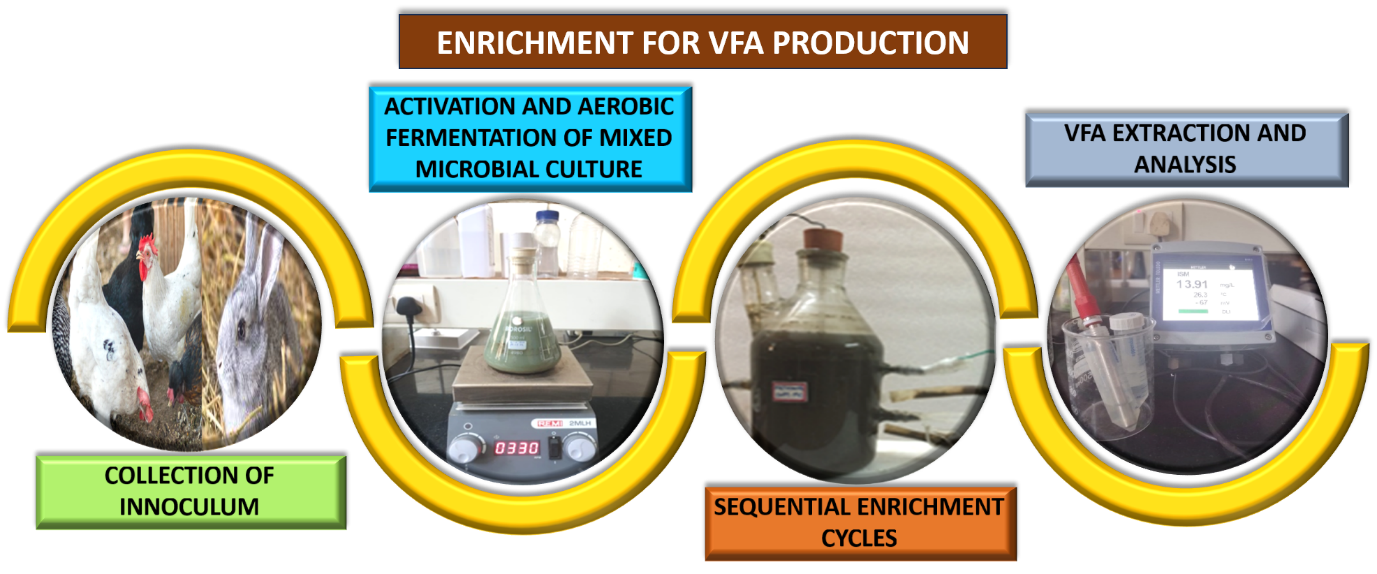

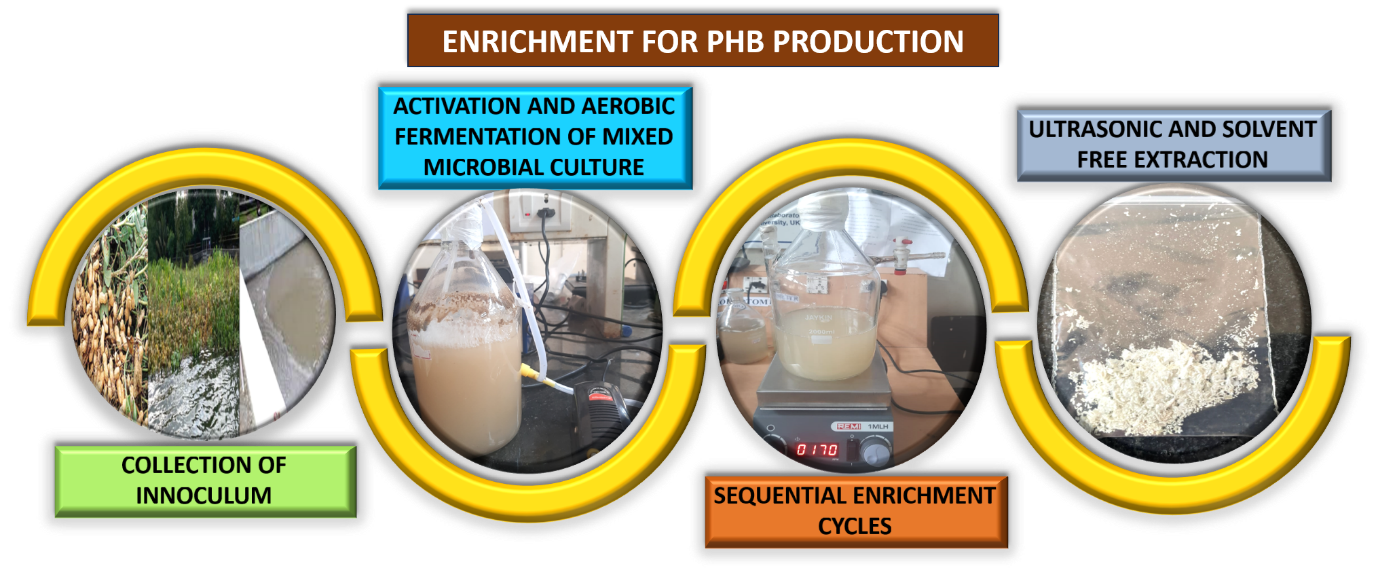
**

**Figure 1: Enrichment process for PHB and VFA production**


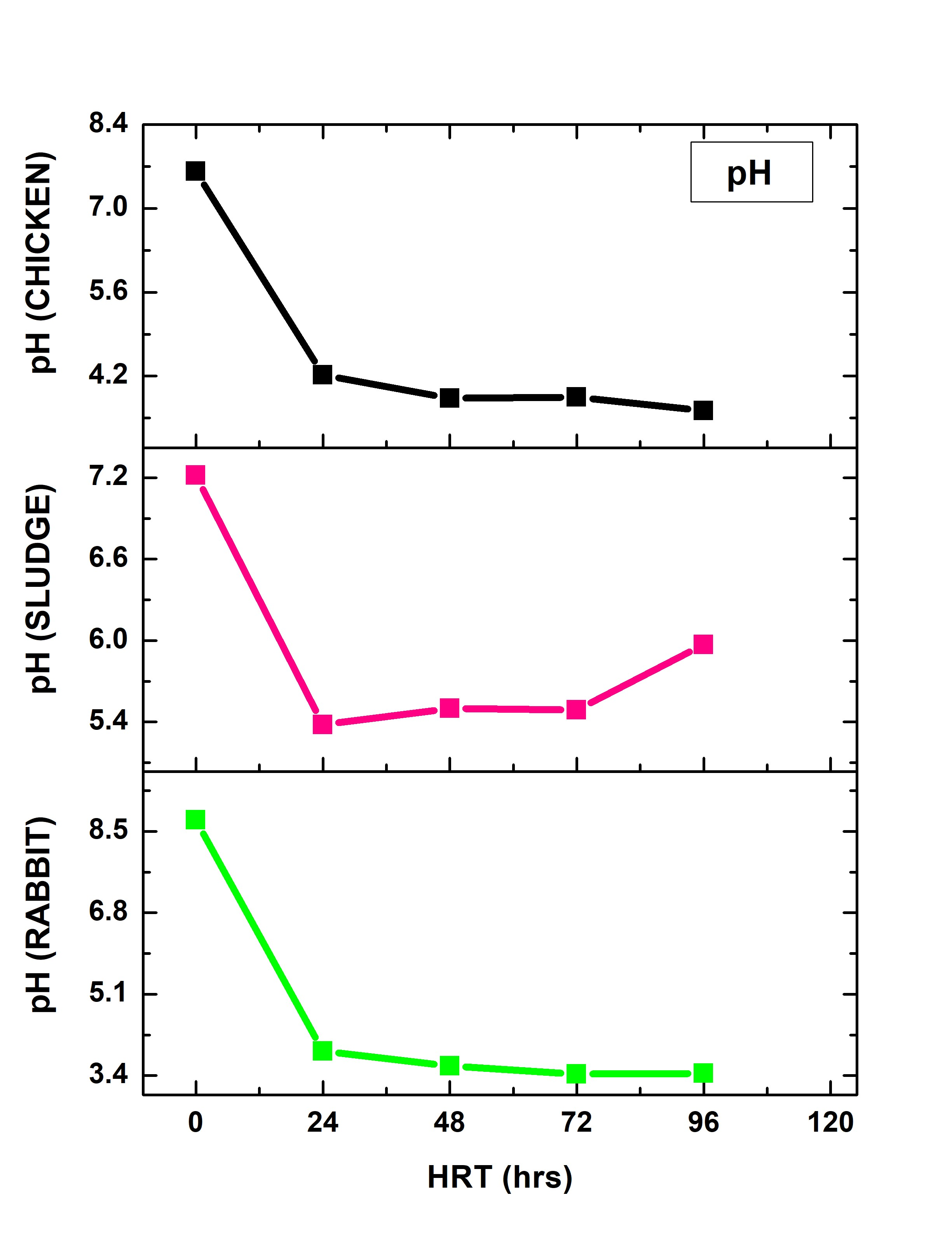


**Figure 2:Activation for MMC from chicken, sludge and rabbit sample**

**List of tables in supplementary:**

**Table-1: ANOVA One Way VFA CONCNTRATION**

**Table-2: ANOVA One Way Descriptive Statistics PHB CONCENTRATION**

**Table-3 : Two way ANOVA CO2 reduction**

**Table-4: Individual sample accessions for SRA**

**Table-5: Input and output carbon ledger**

**Table-1: ANOVA One Way VFA CONCNTRATION**

Descriptive Statistics

|  | Sample Size | Mean | Standard Deviation | SE of mean |
| --- | --- | --- | --- | --- |
| AC | 3 | 2.01667 | 0.16073 | 0.0928 |
| RF | 3 | 2.30667 | 0.08145 | 0.04702 |
| CF | 3 | 3.24333 | 0.1115 | 0.06438 |
| BLEND | 3 | 3.4667 | 0.19858 | 0.11465 |

One Way ANOVA

|  | DF | Sum of Squares | Mean Square | F Value | Prob>F |
| --- | --- | --- | --- | --- | --- |
| Model | 3 | 4.4731 | 1.49103 | 70.72095 | 4.22743E-6 |
| Error | 8 | 0.16867 | 0.02108 |  |  |
| Total | 11 | 4.64177 |  |  |  |

Fit Statistics

| R-Square | Coeff Var | Root MSE | Data Mean |
| --- | --- | --- | --- |
| 0.96366 | 0.05264 | 0.1452 | 2.75833 |

**Table-2: ANOVA One Way Descriptive Statistics PHB CONCENTRATION**

|  | Sample Size | Mean | Standard Deviation | SE of mean |
| --- | --- | --- | --- | --- |
| VFA-PHB | 3 | 6.50667 | 0.3807 | 0.2198 |
| CO2-PHB | 3 | 5.02667 | 0.17926 | 0.10349 |

One Way ANOVA

|  | DF | Sum of Squares | Mean Square | F Value | Prob>F |
| --- | --- | --- | --- | --- | --- |
| Model | 1 | 1.49103 | 3.2856 | 37.11145 | 0.00367 |
| Error | 4 | 0.354153 | 0.08853 |  |  |
| Total | 5 | 3.63973 |  |  |  |

Fit Statistics

| R-Square | Coeff Var | Root MSE | Data Mean |
| --- | --- | --- | --- |
| 0.9027 | 0.0516 | 0.29755 | 5.76667 |

**Table-3 : Two way ANOVA CO2 reduction**

|  | DF | Sum of Squares | Mean Square | F Value | Prob>F |
| --- | --- | --- | --- | --- | --- |
| Culture type | 2 | 732.90621 | 366.45311 | 10.41113 | 0.0017 |
| Enrichment status | 1 | 9709.21125 | 9709.21125 | 9709.21125 | 1.31298E-10 |
| Model | 3 | 10442.11746 | 3480.70582 | 98.8887 | 1.16231E-9 |
| Error | 14 | 492.77503 | 35.19822 |  |  |
| Corrected total | 17 | 35.19822 |  |  |  |

**Table-4: Individual sample accessions for SRA**

| **Accession** | **Sample name** | **Files** | **Title** |
| --- | --- | --- | --- |
| [**SRR34900012**](https://dataview.ncbi.nlm.nih.gov/object/SRR34900012) | [**Rabbit feaces**](https://dataview.ncbi.nlm.nih.gov/object/59532144) | - **BKMTG3150-IshaBRABBIT16S_1.fastq.gz** - **BKMTG3150-IshaBRABBIT16S_2.fastq.gz** | **Rabbit02feaces** |
| [**SRR34900013**](https://dataview.ncbi.nlm.nih.gov/object/SRR34900013) | [**Anaerobic waste water sludge**](https://dataview.ncbi.nlm.nih.gov/object/59532143) | - **BKMTG3150-IshaDANAEROBICSLUDGE16S_1.fastq.gz** - **BKMTG3150-IshaDANAEROBICSLUDGE16S_2.fastq.gz** | **Anaerobic01sludge** |
| [**SRR34900011**](https://dataview.ncbi.nlm.nih.gov/object/SRR34900011) | [**Chicken feaces**](https://dataview.ncbi.nlm.nih.gov/object/59532145) | - **BKMTG3150-IshaCCHICKEN16S_1.fastq.gz** - **BKMTG3150-IshaCCHICKEN16S_2.fastq.gz** | **Chicken03feaces** |
| [**SRR34900009**](https://dataview.ncbi.nlm.nih.gov/object/SRR34900009) | [**Aerobic_waste_water_sludge_acetate**](https://dataview.ncbi.nlm.nih.gov/object/59532147) | - **BKMTG3150- sodium acetate16S_1.fastq.gz** - **BKMTG3150- sodium acetate16S_2.fastq.gz** | **Carbon05acetate** |
| [**SRR34900010**](https://dataview.ncbi.nlm.nih.gov/object/SRR34900010) | [**Aerobic_waste_water_sludge_bicarbonate**](https://dataview.ncbi.nlm.nih.gov/object/59532146) | - **BKMTG3150- sodium bicarnonate16S_1.fastq.gz** - **BKMTG3150- sodium bicarnonate16S_2.fastq.gz** | **Carbon04bicarbonate** |

**Table-5: Input and output carbon ledger**

|  | AC | RF | CF |
| --- | --- | --- | --- |
| Components | Carbon (g C/L) | Carbon (g C/L) | Carbon (g C/L) |
| Input - NaHCO₃ | 0.858 g C/L | 0.858 g C/L | 0.858 g C/L |
| Input - CO₂ (dissolved) | 0.048 g C/L | 0.054 g C/L | 0.062 g C/L |
| Input - Inoculum biomass | 450 g C/L | 0.400 g C/L | 0.340 g C/L |
| Input - Total Inputs | 1.356 g C/L | 1.312 g C/L | 1.260 g C/L |
| Output - Acetate | 0.480 g C/L | 0.600 g C/L | 0.730 g C/L |
| Output - Butyrate | 0.324 g C/L | 0.410 g C/L | 0.450 g C/L |
| Output - Biomass (output) | 0.540 g C/L | 0.480 g C/L | 0.408 g C/L |
| Output - Residual CO₂ | 0.025 g C/L | 0.025 g C/L | 0.025 g C/L |
| Output - Total Outputs | 1.369 g C/L | 1.515 g C/L | 1.613 g C/L |
| Output - Closure % | 101.00% | 115.50% | 128.00% |

|  | PHB REACTOR |
| --- | --- |
| Component | Carbon (g C/L) |
| Input - NaHCO₃ | 0.858 g C/L |
| Input - CO₂ (dissolved) | 0.052 g C/L |
| Input - Biomass (input) | 1.29 g C/L |
| Input - Total Inputs | 1.125 g C/L |
| Output - Biomass (output) | 0.3455 g C/L |
| Output - PHB | 0.220 g C/L |
| Output - Other metabolites | 0.565 g C/L |
| Output - Residual CO₂ | 0.025 g C/L |
| Output - Total Outputs | 1.1555 g C/L |
| Output - Closure % | 102.70% |
